# Total Synthesis of the Racemate of Laurolitsine

**DOI:** 10.3390/molecules29030745

**Published:** 2024-02-05

**Authors:** Mingyu Cao, Yiming Wang, Yong Zhang, Caiyun Zhang, Niangen Chen, Xiaopo Zhang

**Affiliations:** 1 Key Laboratory of Tropical Translational Medicine of Ministry of Education, Hainan Key Laboratory for Research and Development of Tropical Herbs, School of Pharmacy, Hainan Medical University, Haikou 571199, China; 13178932187@163.com (M.C.); wym1356046750@163.com (Y.W.); hy0206162@hainmc.edu.cn (Y.Z.); 2 Research Center for Drug Safety Evaluation of Hainan Province, Hainan Medical University, Haikou 571101, China; zhangcaiyun-tracy@163.com

**Keywords:** total synthesis, laurolitsine, aporphine alkaloid, synthetic route

## Abstract

The total synthesis of laurolitsine was achieved for the first time. This reaction was accomplished in 14 steps with a 2.3% yield (this was calculated using 3-hydroxy-4-methoxybenzaldehyde as the starting material) starting from two simple materials, 3-hydroxy-4-methoxybenzaldehyde and 2-(3-hydroxy-4-methoxyphenyl)acetic acid, and the longest linear sequence consisted of 11 steps. The key steps included an electrophilic addition reaction in which a nitro group was reduced to an amino group using lithium tetrahydroaluminum and a Pd-catalyzed direct biaryl coupling reaction. In this paper, many of the experimental steps were optimized, and an innovative postprocessing method in which 2-(3-(benzyloxy)-4-methoxyphenyl)ethanamine is salted with oxalic acid was proposed.

## 1. Introduction

Laurolitsine is an alkaloid isolated from natural plants such as *Litsea glutinosa* that exhibits potent antidiabetic effects and hypoglycemic activity in vivo and has wide application prospects in the clinic [1,2]. Laurolitsine was first discovered and named by Tatsuo Nakasato and Shozo Nomura from the leaves of *Neolitsea sericea* (Blume) Koidz. in 1959 [3]. Sun et al. obtained laurolitsine by efficient isolation from the chloroform extract of *Litsea cubeba* [4]. Through a series of experiments, Zhang et al. confirmed that laurolitsine, which is abundant in *Litsea glutinosa* bark, can exhibit potent antidiabetic effects with hypoglycemic activity in vivo. Laurolitsine improved insulin resistance, glucose tolerance and lipid metabolism; protected liver, renal and pancreatic functions; and promoted weight loss in db/db mice [1].

However, since laurolitsine must be isolated and extracted from plants, the cycle is long, and low yields are obtained; thus, the price of this material is relatively high, which greatly limits the development of related experiments on laurolitsine [4]. At present, there are no reports on the total synthesis of laurolitsine. Therefore, exploring a new synthesis route is imperative, and reasonable and efficient chemical synthesis methods for obtaining laurolitsine quickly and in large quantities are very important for related research.

**Figure 1:**
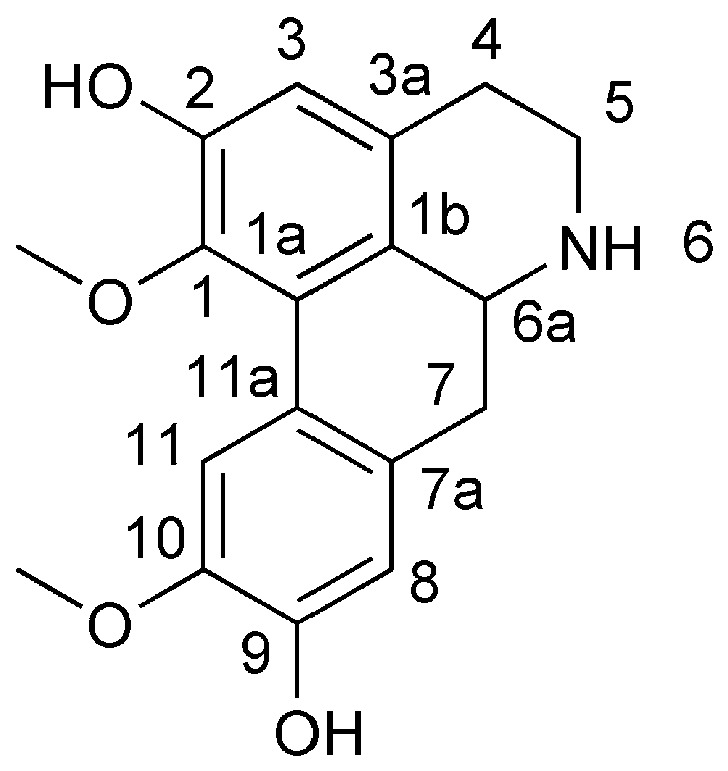
The structure of laurolitsine.

Laurolitsine is an aporphine alkaloid that has a special biphenyl tetracyclic structure with a chiral carbon atom at the 6a position (Figure 1) and a wide range of physiological activities due to its different oxidation states and substituents [5,6,7,8,9,10]. Due to their remarkable pharmacological effects, aporphine alkaloids have attracted widespread attention in organic synthesis. However, directly synthesizing aporphine alkaloids is challenging due to their special benzene ring structure. The 1-benzyl-substituted tetrahydroisoquinoline can be used as a basic skeleton for the biomimetic synthesis of aporphine alkaloids. Therefore, establishing this parent nucleus structure is fundamental to the synthesis of aporphine alkaloids [11].

Lafrance et al. reported that a variety of aporphine alkaloids with 2-position substitutions can be directly synthesized using a Pd-catalyzed arylation reaction [12]. This breakthrough not only highlights the utility of direct arylation in target-oriented synthesis reactions but also allows the synthesis of various compounds with different substituents. Researchers have used the same approach for the enantioselective synthesis of pronuciferine and nuciferin [11]. Gao et al. described a highly efficient and practical multicomponent one-pot reaction. This reaction represents a streamlined pathway for synthesizing functionalized 1,2-dihydroisoquinolines, showcasing the versatility of multicomponent reactions in organic synthesis [13]. Under different reaction conditions, a novel series of aporphine analogs were finally accomplished through intramolecular phenol ortho-arylation using Pd-mediated catalysis [14,15,16,17,18]. However, the operation is complex, and most steps require silica gel column chromatography. The purpose of this study was to synthesize total amounts of laurolitsine for the first time and optimize the reaction steps. An innovative postprocessing method in which 2-(3-(benzyloxy)-4-methoxyphenyl)ethanamine is salted with oxalic acid is also proposed, which greatly reduces the time cost and increases the yield of intermediates.

## 2. Results and Discussion

### 2.1. Retrosynthetic Analysis of Laurolitsine

**Scheme 1:**
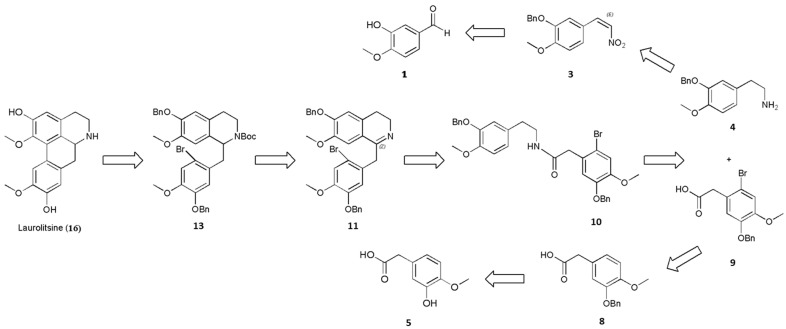
Retrosynthetic analysis of laurolitsine.

As shown in Scheme 1, the steps for the synthesis of laurolitsine (**16**) begins with the N-*t*-butyloxy carbonyl (Boc)-protected carbamate **13** and uses Pd-catalyzed direct arylation and deprotection reaction. N-Boc-protected carbamate **13** is then generated from cyclized imine **11** through a reduction reaction and protection with a Boc group. Cylized imine **11** is generated from amide **10** through the Bischler–Napieralski reaction under acidic conditions. Eventually, amide **10** could be prepared from 2-(3-(benzyloxy)-4-methoxyphenyl)ethanamine (**4**) coupled with 2-(5-(benzyloxy)-2-bromo-4-methoxyphenyl)acetic acid (**9**) under standard peptide-coupling conditions.

Compounds **4** and **9** were synthesized by two different routes. Compound **3** was synthesized through a benzylation reaction and a nitration reaction, and **4** was subsequently produced by the reduction of **3**. In relation to another reaction route, **9** was produced from **8** through substitution with Br_2_. Finally, **8** was obtained by esterification, benzylation, and hydrolysis.

### 2.2. Facile Construction of the Laurolitsine Skeleton

**Scheme 2:**
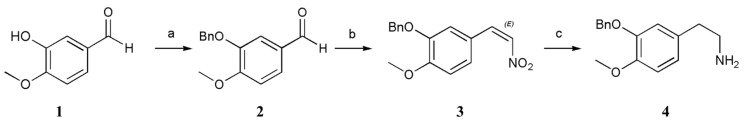
Conditions: (a) K_2_CO_3_, BnCl, CH_3_CN, 82 °C, 83%; (b) NH_4_OAc, CH_3_NO_2_, HOAc, 118 °C, 89%; (c) LiAlH_4_, THF, 30 °C, 50%.

The protection of **1** with a benzyl (Bn) group furnished **2**. Then, **3** was prepared via the Henry reaction and elimination reaction by adding **2**, ammonium acetate, and nitromethane to an acetic acid solvent at 118 °C [19,20,21]. First, we wanted to complete the reaction by increasing the amount of nitromethane and ammonium acetate, but despite our efforts to perform many experiments, the effect was not good, the product was sticky and difficult to separate and filter, and the yield and purity were poor. After many experimental alterations, we finally determined the reaction conditions (CH_3_NO_2_ (4 equiv.), NH_4_OAc (1.3 equiv.), HOAc, 118 °C). The viscosity of the product may be related to the amount of ammonium acetate. If the amount of ammonium acetate is too high, side reactions increase and intermediate **3** becomes viscous and difficult to filter. A small amount of ammonium acetate causes the reaction to be incomplete. Regarding the next step of reduction, we attempted to reduce Pd/C, iron powder, sodium sulfide and sodium borohydride and did not achieve good results; subsequently, we succeeded by adding the strong reductant LiAlH_4_, which was converted to substituted phenyl ethanamine **4** via reduction. LiAlH_4_ was selected to reduce the double bond and nitro group simultaneously. A much lower yield was obtained when LiAlH_4_ was added at ambient temperature due to the generation of impurities including two compounds that only reduced the nitro group but not the double bond and only reduced the double bond but not the nitro group. Purifying the product was difficult because reduction of the double bond and nitro group was incomplete, and the polarity difference between the two impurities was very small [19,20,21]. In terms of the feeding temperature, reaction temperature, and reductant ratio, we performed a large number of experiments and ultimately determined the most suitable reaction conditions (LiAlH_4_ (4 equiv.), THF, 0 → 35 °C, THF = tetrahydrofuran). A method involving salt formation between oxalic acid and amino groups was used for purification. Compound **4** was combined with oxalic acid in methanol to form oxalate, after which the salt was hydrolyzed with sodium hydroxide solution, which achieved high purity and reduced the number of tedious purification steps. The specific method is described in detail later (Scheme 2).

**Scheme 3:**
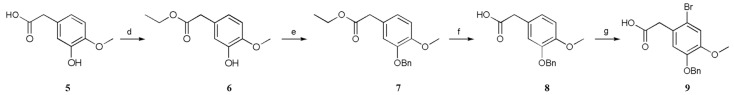
Conditions: (d) EtOH, H_2_SO_4_, 78 °C, 90%; (e) K_2_CO_3_, BnCl, CH_3_CN, 82 °C, 82%; (f) NaOH, EtOH, 78 °C, 93%; (g) Br_2_, NaOAc, HOAc, 25 °C, 61%.

First, **5** was esterified with ethanol to furnish **6**, and then **7** was formed by a benzylation reaction. Next, **8** was obtained by hydrolysis with sodium hydroxide solution, and **9** was obtained by reaction with Br_2_, acetic acid and sodium acetate [15]. Notably, this reaction requires Br_2_ to be slowly added to the system at the end; otherwise, the reaction will not be complete (Scheme 3).

We thus attempted to repeat the work reported by Sharma et al. [18] by coupling 2-(3-(benzyloxy)-4-methoxyphenyl)ethanamine (**4**) with 2-(5-(benzyloxy)-2-bromo-4-methoxyphenyl)acetic acid (**9**) under standard peptide-coupling conditions to afford amide **10** (1,1′-carbonyldiimidazole, THF, RT, 20 h). Unfortunately, we could not observe any reactions between **4** and **9**. We varied the concentration of the reaction mixture and the number of equivalents of each reagent, and no good effects were observed. Considering that **4** contains a benzyl group and has a large steric hindrance, we subsequently changed the solvent to xylene and dehydrated and condensed the mixture with an oil–water separator at 135 °C; however, no satisfactory results were obtained. Finally, we tried to obtain **10** successfully by using DMF, HOBt and EDC. The use of the dehydrating condensation agent EDC results in mild reaction conditions and an easy process.

**Scheme 4:**
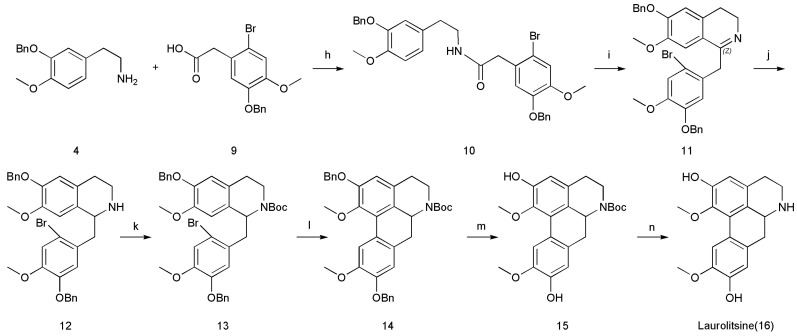
Conditions: (h) DMF, HOBt, EDC, 74%; (i) POCl_3_, CH_3_CN, 82 °C, 71%; (j) NaBH_4_, EtOH, 25 °C, 72%; (k) (Boc)_2_O, Et_3_N, CH_2_Cl_2_, 25 °C, 80%; (l) Cs_2_CO_3_, Pd(OAc)_2_, (t-Bu)_2_PMeHBF_4_, 1,4-dioxane, 100 °C, 53%; (m) Pd/C, THF, HOAc, 25 °C, 82%; (n) ZnBr_2_, CH_2_Cl_2_, 25 °C, 49%.

Amide **10** was subjected to the Bischler–Napieralski reaction [22] to afford cyclized imine **11**, which was subjected to NaBH_4_-mediated reduction without further purification to furnish secondary amine **12**. The protection of secondary amine **12** with a tert-butoxycarbonyl (Boc) group furnished N-Boc-protected **13**. Then, **14** was prepared by a Pd-catalyzed direct biaryl coupling methodology [23,24,25]. If the reaction was carried out at a lower temperature for a long time, debrominated byproducts appeared. The selection of ligands was also particularly important, because different ligands had different stability, which will greatly affect the yield. In terms of the choice of solvent, we chose 1,4-dioxane. Compared with DMA, DMF or DMSO, our advantage was that we can choose to directly steam the solvent after the reaction was complete, avoiding the product loss and tedious operation steps brought by extraction. We tested many reaction conditions and determined the most suitable reaction conditions (Pd(OAc)_2_ (0.2 equiv.), Cs_2_CO_3_ (3 equiv.), (t-Bu)_2_PMeHBF_4_ (0.4 equiv.), 1,4-dioxane, 101 °C, (t-Bu)_2_PMeHBF_4_ = di-tert-butylmethylphosphine tetrafluor). It was worth noting that the reaction conditions were harsh and required very strict vacuum and nitrogen protection. The synthesis of **15** was achieved by deprotecting the benzyl (Bn) group of **14** by using Pd/C under a hydrogen atmosphere. Finally, the synthesis of laurolitsine (**16**) was achieved by deprotecting the Boc group of **15** by using anhydrous ZnBr_2_ [18]. Spectral data, including ^1^H NMR, ^13^C NMR, were collected for both the natural and synthetic sample (**16**) and found to be in good agreement (Table 1) [4]. All these compounds were well characterized by using ^1^H NMR, ^13^C NMR, and high-resolution (HR) ESI-MS, as showed in Supplementary Materials (Scheme 4).

## 3. Materials and Methods

### 3.1. General Experimental Details

All chemicals were purchased from commercial sources. Silica gel (200–300 mesh, Qingdao Marine Chemistry Co. Ltd., Qingdao, Tsingtao, China) was used for column chromatography. Reactions were monitored by thin-layer chromatography (TLC). Silica gel plates (Qingdao Marine Chemistry Co. Ltd., GF254, 0.20–0.25 mm) were used for the TLC analyses, which were visualized under model ZF-20D ultraviolet analyzing equipment (Shanghai Baoshan Gucun Photoelectricity Instrument, Shanghai, China) at 254 nm. ^1^H and ^13^C NMR experiments were performed on a JNM-ECZ400S NMR Spectrometer (Nippon Electric Company Limited, Tokyo, Japan) at 400 MHz for ^1^H NMR and 100 MHz for ^13^C NMR. Chemical shifts (*δ*) are given in parts per million (ppm), and coupling constants are given in Hz. The multiplicity of ^1^H NMR signals is reported as follows: s = singlet, d = doublet, t = triplet, q = quartet, m = multiplet, br = broad, dd = doublet of doublets. The HRMS (ESI) spectroscopic data were obtained from an Agilent 1290II Mass Spectrometer (Agilent Technologies Inc., Santa Clara, CA, USA).

### 3.2. Synthesis and Characterization of the Compounds

#### 3.2.1. 3-(Benzyloxy)-4-methoxybenzaldehyde (**2**)

A solution of 3-hydroxy-4-methoxybenzaldehyde (**1**; 1.52 g, 10.0 mmol, 1 equiv.), BnCl (1.52 g, 12.0 mmol, 1.2 equiv.), K_2_CO_3_ (4.15 g, 30.0 mmol, 3 equiv.), and KI (4.15 mg, 0.025 mmol, 0.025 equiv.) in anhydrous CH_3_CN (10 mL) was stirred at 82 °C for 2 h. The mixture was then cooled to 20–30 °C, after which the precipitate was filtered off. The solvent was removed under reduced pressure, and the obtained residue was dissolved in EtOAc (20 mL) and washed sequentially with Sad. NaHCO_3_ solution (2 × 10 mL), water (2 × 10 mL), and brine (2 × 10 mL). The organic layer was dried (anhydrous NaSO_4_) and concentrated under reduced pressure. The resultant crude products were purified by recrystallization (hexane), which furnished 3-(benzyloxy)-4-methoxybenzaldehyde (**2**) (83% yield).

3-(Benzoxy)-4-methoxybenzaldehyde (**2**): white solid; Rf (Hexane/EtOAc 80:20) = 0.62; purification by flash column chromatography (deactivated silica gel, Hexane/EtOAc 90:10); ^1^H-NMR (400 MHz, CDCl_3_): *δ* = 9.80 (s, 1H), 7.46–7.44 (m, 4H), 7.38–7.34 (m, 2H), 7.32 (m, 1H), 7.00 (d, *J* = 8.8 Hz, 1H), 5.17 (s, 2H), 3.94 (s, 3H); ^13^C-NMR (100 MHz, CDCl_3_): *δ* = 190.97, 155.15, 148.79, 136.38, 130.07, 128.75 (2C), 128.23, 127.59 (2C), 127.01, 111.44, 110.88, 70.93, 56.27. HRMS (ESI) calculated for C_15_H_15_O_3_^+^ [M + H]^+^ 243.1016, found 243.1013.

#### 3.2.2. 2-(Benzyloxy)-1-methoxy-4-((E)-2-nitrovinyl)benzene (**3**)

A solution of 3-(benzyloxy)-4-methoxybenzaldehyde (**2**; 2.42 g, 10.0 mmol, 1 equiv.), NH_4_OAc (1.00 g, 13.0 mmol, 1.3 equiv.), and CH_3_NO_2_ (2.44 g, 40.0 mmol, 4 equiv.) in anhydrous HOAc (10 mL) was stirred at 118 °C for 4 h. The mixture was then cooled to 20–30 °C, after which the precipitate was filtered off. The precipitate was washed with water to neutral, which furnished 2-(benzyloxy)-1-methoxy-4-((E)-2-nitrovinyl)benzene (**3**) (89% yield).

2-(Benzoxy)-1-methoxy-4-((E)-2-nitrovinyl)benzene (**3**): yellow solid; Rf (Hexane/EtOAc 80:20) = 0.66; purification by flash column chromatography (deactivated silica gel, Hexane/EtOAc 90:10) ^1^H-NMR (400 MHz, CDCl_3_): *δ* = 7.89 (d, *J* = 13.6 Hz, 1H), 7.42–7.30 (m, 6H), 7.15–7.13 (m, 1H), 7.01 (s, 1H), 6.91–6.89 (m, 1H), 5.14 (s, 2H), 3.91 (s, 3H); ^13^C-NMR (100 MHz, CDCl_3_): *δ* = 153.56, 148.68, 139.42, 136.37, 135.20, 128.83 (2C), 128.32, 127.43 (2C), 125.02, 122.74, 113.22, 111.83, 71.25, 56.20. HRMS (ESI) calculated for C_16_H_16_NO_4_^+^ [M + H]^+^ 286.1074, found 286.1072.

#### 3.2.3. 2-(3-(Benzyloxy)-4-methoxyphenyl)ethanamine (**4**)

A flask containing anhydrous THF (15 mL) was cooled to 0 °C under N_2_, after which LiAlH_4_ (1.52 g, 40.0 mmol, 4 equiv.) was added cautiously. A solution of 2-(benzyloxy)-1-methoxy-4-((E)-2-nitrovinyl)benzene (**3**; 2.85 g, 10.0 mmol, 1 equiv.) in anhydrous THF (30 mL) was added dropwise at 35 °C. The temperature of the system was controlled at 35 °C under N_2_ for 4 h. Upon completion, the reaction mixture was cooled to 0 °C and slowly quenched with water (6 mL). After 15 min, 15% (*w*/*w*) aq. NaOH (3 mL) was added. The resultant mixture was stirred for 30 min at 20–30 °C, and the mixture was filtered through a Celite pad with anhydrous MgSO_4_. The filtrate was concentrated, and the residue was purified as follows: methanol (15 mL) was added to the residue and allowed to dissolve completely by stirring. Anhydrous oxalic acid (1.05 g) was also added at 45 °C. Once completely dissolved, ethyl acetate (50 mL) was introduced into the mixture, and a significant amount of white solid was precipitated. The reaction was stirred for 2 h before cooling it down to 20–30 °C, after which we filtered the solution and air dried the obtained filter cake. Next, we adjusted the pH value to approximately 9–10 by adding a solution containing 30% (*w*/*w*) aq. NaOH (40 mL) while continuously stirring magnetically for 2 h. The solid was filtered, and then we beat the filter cake with water (50 mL) for 2 h before filtering it once more. Finally, the resulting filter cake was dried by air, which furnished the 2-(3-(benzyloxy)-4-methoxyphenyl)ethanamine (**4**) (50% yield).

2-(3-(Benzoxy)-4-methoxyphenyl)ethanamine (**4**): white solid; Rf (MeOH/CH_2_Cl_2_ 90:10, 0.02% NH_3_·H_2_O) = 0.56; purification by flash column chromatography (deactivated silica gel, MeOH/CH_2_Cl_2_ 95:5) ^1^H-NMR (400 MHz, CDCl_3_): *δ* = 7.39–7.37 (m, 2H), 7.30–7.26 (m, 2H), 7.23–7.20 (m, 1H), 7.96 (d, *J* = 8.0 Hz, 1H), 6.70–6.66 (m, 2H), 5.06 (s, 2H), 3.76 (s, 3H); ^13^C-NMR (100 MHz, CDCl_3_): *δ* = 148.30, 148.09, 137.28, 132.27, 128.58 (2C), 127.90, 127.48 (2C), 121.56, 115.14, 112.10, 71.07, 56.10, 43.47, 39.16. HRMS (ESI) calculated for C_16_H_20_NO_2_^+^ [M + H]^+^ 258.1489, found 258.1489.

#### 3.2.4. Ethyl 2-(3-Hydroxy-4-methoxyphenyl)acetate (**6**)

A solution of 2-(3-hydroxy-4-methoxyphenyl)acetic acid (**5**; 1.82 g, 10.0 mmol, 1 equiv.) and H_2_SO_4_ (1.83 g, 1 mL, 18.7 mmol, 1.87 equiv.) in anhydrous EtOH (10 mL) was stirred at 78 °C for 4 h. The mixture was then cooled to 20–30 °C. The solvent was removed under reduced pressure, and the obtained residue was dissolved in EtOAc (10 mL) and washed sequentially with Sad. NaHCO_3_ solution (2 × 2 mL), water (2 × 5 mL), and brine (2 × 5 mL). The organic layer was dried (anhydrous NaSO_4_) and concentrated under reduced pressure, which furnished ethyl 2-(3-hydroxy-4-methoxyphenyl)acetate (**6**) (90% yield).

Ethyl 2-(3-hydroxy-4-methoxyphenyl)acetate (**6**): colorless oil; Rf (Hexane/EtOAc 35:65) = 0.66; purification by flash column chromatography (deactivated silica gel, Hexane/EtOAc 60:40) ^1^H-NMR (400 MHz, CDCl_3_): *δ* = 6.78–6.74 (m, 1H), 6.61–6.57 (m, 2H), 6.49 (br, 1H), 4.01–3.95 (m, 2H), 3.59–3.55 (m, 3H), 3.39–3.35 (m, 2H), 1.10–1.02 (m, 3H); ^13^C-NMR (100 MHz, CDCl_3_): *δ* = 172.10, 146.14, 145.82, 127.17, 120.70, 115.88, 111.06, 60.81, 55.71, 40.58, 13.99. HRMS (ESI) data were calculated for C_11_H_14_O_4_Na^+^ [M + Na]^+^ 233.0784, found 233.0788.

#### 3.2.5. Ethyl 2-(3-(Benzyloxy)-4-methoxyphenyl)acetate (**7**)

A solution of ethyl 2-(3-hydroxy-4-methoxyphenyl)acetate (**6**; 2.1 g, 10.0 mmol, 1 equiv.), BnCl (1.52 g, 12.0 mmol, 1.2 equiv.), K_2_CO_3_ (4.15 g, 30.0 mmol, 3 equiv.), and KI (4.15 mg, 0.025 mmol, 0.025 equiv.) in anhydrous CH_3_CN (10 mL) was stirred at 82 °C for 3 h. The mixture was then cooled to 20–30 °C, after which the precipitate was filtered off. The solvent was removed under reduced pressure, and the obtained residue was dissolved in EtOAc (20 mL) and washed sequentially with Sad. NaHCO_3_ solution (2 × 10 mL), water (2 × 10 mL), and brine (2 × 10 mL). The organic layer was dried (anhydrous NaSO_4_) and concentrated under reduced pressure. The resultant crude products were purified by recrystallization (hexane), which furnished ethyl 2-(3-(benzyloxy)-4-methoxyphenyl)acetate (**7**) (82% yield).

2-(3-(Benzoxy)-4-methoxyphenyl)acetate (**7**): white solid; Rf (Hexane/EtOAc 35:65) = 0.66; purification by flash column chromatography (deactivated silica gel, Hexane/EtOAc 60:40) ^1^H-NMR (400 MHz, CDCl_3_): *δ* = 7.45–7.27 (m, 5H), 6.87–6.83 (m, 3H), 5.13 (s, 1H), 4.11 (q, 2H), 3.85 (s, 3H), 3.49 (s, 2H), 1.21 (t, 3H); ^13^C-NMR (100 MHz, CDCl_3_): *δ* = 171.88, 148.93, 148.24, 137.20, 128.62 (2C), 127.93, 127.47 (2C), 126.68, 122.13, 115.18, 111.95, 71.09, 60.88, 56.13, 40.99, 14.28. HRMS (ESI) calculated for C_18_H_21_O_4_^+^ [M + H]^+^ 301.1434, found 301.1436.

#### 3.2.6. 2-(3-(Benzyloxy)-4-methoxyphenyl)acetic Acid (**8**)

A solution of ethyl 2-(3-(benzyloxy)-4-methoxyphenyl)acetate (**7**; 3.00 g, 10.0 mmol, 1 equiv.) and 20% (*w*/*w*) aq. NaOH (40 mL) in anhydrous EtOH (15 mL) was stirred at 78 °C for 3 h. The mixture was then cooled to 20–30 °C. The solvent was removed under reduced pressure, and the obtained residue was dissolved in EtOAc (10 mL) and water (10 mL). Following this, the 10% (*w*/*w*) aq. HCl was added to adjust the pH to 2–3. The precipitate was filtered off. The precipitate was washed with water to neutral, which furnished 2-(3-(benzyloxy)-4-methoxyphenyl)acetic acid (**8**) (93% yield).

2-(3-(Benzoxy)-4-methoxyphenyl)acetic acid (**8**): white solid; Rf (Hexane/EtOAc 80:20) = 0.53; purification by flash column chromatography (deactivated silica gel, Hexane/EtOAc 50:50) ^1^H-NMR (400 MHz, CDCl_3_): *δ* = 7.43–7.27 (m, 5H), 6.84–6.83 (m, 3H), 5.11 (s, 2H), 3.85 (s, 3H), 3.52 (s, 2H); ^13^C-NMR (100 MHz, CDCl_3_): *δ* = 177.96, 149.13, 148.27, 137.04, 128.63 (2C), 127.98, 127.57 (2C), 125.75, 122.29, 115.34, 111.96, 71.15, 56.12, 40.60. HRMS (ESI) calculated for C_16_H_16_O_4_Na^+^ [M + Na]^+^ 295.0941, found 295.0944.

#### 3.2.7. 2-(5-(Benzyloxy)-2-bromo-4-methoxyphenyl)acetic Acid (**9**)

Bromine (11.2 mmol, 1.12 equiv.) was cautiously added to a solution of 2-(3-(benzyloxy)-4-methoxyphenyl)acetic acid (**8**; 2.72 g, 10.0 mmol, 1 equiv.) and anhydrous sodium acetate (1.36 g, 34.0 mmol, 3.4 equiv.) in acetic acid (15 mL), and the mixture was stirred for 1 h at 20–30 °C. The precipitate was filtered off and washed with water to neutral pH, which furnished 2-(5-(benzyloxy)-2-bromo-4-methoxyphenyl)acetic acid (**9**) (61% yield).

2-(5-(Benzoxy)-2-bromo-4-methoxyphenyl)acetic acid (**9**): white solid; Rf (Hexane/EtOAc 65:35) = 0.46; purification by flash column chromatography (deactivated silica gel, Hexane/EtOAc 85:15) ^1^H-NMR (400 MHz, CDCl_3_): *δ* = 8.02 (br, 1H), 7.41–7.29 (m, 5H), 7.05 (s, 1H), 6.82 (s, 1H), 5.09 (s, 2H), 3.84 (s, 3H), 3.70 (s, 2H); ^13^C-NMR (100 MHz, CDCl_3_): *δ* = 176.87, 149.80, 147.63, 136.56, 128.71 (2C), 128.18, 127.56 (2C), 125.25, 116.74, 116.04, 115.80, 71.39, 56.32, 40.85. HRMS (ESI) calculated for C_16_H_15_BrO_4_Na^+^ [M + Na]^+^ 373.0046, found 373.0050.

#### 3.2.8. N-(3-(Benzoxy)-4-methoxyphenethyl)-2-(5-(benzyloxy)-2-bromo-4-methoxyphenyl)acetamide (**10**)

Next, 1-hydroxybenzotriazole (2.03 g, 15.0 mmol, 1.5 equiv.) was added to a solution of 2-(3-(benzyloxy)-4-methoxyphenyl)ethanamine (**4**; 2.57 g, 10.0 mmol, 1 equiv.) and 2-(5-(benzyloxy)-2-bromo-4-methoxyphenyl)acetic acid (**9**; 3.69 g, 10.5 mmol, 1.05 equiv.) in dry DMF (50 mL). The reaction mixture was cooled to 0 °C, after which 1-(3-dimethylaminopropyl)-3-ethyl-carbodiimide hydrochloride (2.3 g, 12.0 mmol, 1.2 equiv.) was added. The reaction mixture was warmed slowly to 20–30 °C and then stirred for 4 h before being quenched by the addition of NaHCO_3_ (25 mL, sat., aq.) and extracted with EtOAc (3 × 25 mL). The combined organic layers were washed with brine, dried over MgSO_4_, filtered and concentrated to afford N-(3-(benzyloxy)-4-methoxyphenethyl)-2-(5-(benzyloxy)-2-bromo-4-methoxyphenyl)acetamide (**10**) (74% yield).

N-(3-(benzyloxy)-4-methoxyphenethyl)-2-(5-(benzyloxy)-2-bromo-4-methoxyphenyl)acetamide (**10**): white solid; Rf (Hexane/EtOAc 65:35) = 0.62; purification by flash column chromatography (deactivated silica gel, Hexane/EtOAc 85:15) ^1^H-NMR (400 MHz, CDCl_3_): *δ* = 7.43–7.28 (m, 10H), 6.99 (s, 1H), 6.78 (s, 1H), 6.73 (d, *J* = 8.1 Hz, 1H), 6.66 (d, *J* = 2.0 Hz, 1H), 6.58 (dd, *J* = 8.1, 2.0 Hz, 1H), 5.33 (t, *J* = 5.8 Hz, 1H), 5.08 (s, 2H), 5.06 (s, 2H), 3.82 (s, 3H), 3.81 (s, 3H), 3.49 (s, 2H), 3.37 (q, *J* = 7.0 Hz, 2H), 2.60 (t, *J* = 6.9 Hz, 2H); ^13^C-NMR (100 MHz, CDCl_3_): *δ* = 169.83, 149.73, 148.46, 148.30, 147.81, 137.15, 136.42, 131.05, 128.70 (2C), 128.62 (2C), 128.19, 127.96, 127.54 (2C), 127.47 (2C), 126.49, 121.39, 116.43, 116.11, 115.48, 114.69, 111.97, 71.17, 71.10, 56.29, 56.10, 43.64, 40.72, 34.94, 29.78. HRMS (ESI) data were calculated for C_32_H_33_BrNO_5_^+^ [M + H]^+^ 590.1537, found 590.1538.

#### 3.2.9. 1-(5-(Benzoxy)-2-bromo-4-methoxybenzyl)-6-(benzyloxy)-3,4-dihydro-7-methoxyisoquinoline (**11**)

A solution of N-(3-(benzyloxy)-4-methoxyphenethyl)-2-(5-(benzyloxy)-2-bromo-4-methoxyphenyl)acetamide (**10**; 5.91 g, 10.0 mmol, 1 equiv.) and POCl_3_ (1.84 g, 12.0 mmol, 1.2 equiv.) in anhydrous CH_3_CN (10 mL) was stirred at 82 °C for 4 h. The mixture was then cooled to 20–30 °C and quenched slowly with water (6 mL). The solvent was removed under reduced pressure. The resultant crude products were purified by column chromatography on silica gel (CH_2_Cl_2_), which furnished 1-(5-(benzyloxy)-2-bromo-4-methoxybenzyl)-6-(benzyloxy)-3,4-dihydro-7-methoxyisoquinoline (**11**) (71% yield).

1-(5-(Benzyloxy)-2-bromo-4-methoxybenzyl)-6-(benzyloxy)-3,4-dihydro-7-methoxyisoquinoline (**11**): White solid; Rf (CH_2_Cl_2_/MeOH 95:5) = 0.52; purification by flash column chromatography (deactivated silica gel, CH_2_Cl_2_) ^1^H-NMR (400 MHz, CDCl_3_): *δ* = 7.42–7.18 (m, 10H), 7.03 (s, 1H), 6.90 (s, 1H), 6.79 (s, 1H), 6.62 (s, 1H), 5.15 (s, 2H), 5.00 (s, 2H), 4.04 (s, 2H), 3.82 (s, 3H), 3.79 (s, 3H), 3.59 (t, *J* = 7.5 Hz, 2H), 2.43 (t, *J* = 7.6 Hz, 2H); ^13^C-NMR (100 MHz, CDCl_3_): *δ* = 165.65, 150.04, 148.98, 147.99, 147.70, 136.71, 136.68, 131.43, 129.43, 128.76 (2C), 128.54 (2C), 128.13, 127.88, 127.28 (2C), 127.16 (2C), 121.61, 115.78, 114.87, 114.69, 112.31, 109.75, 70.97, 70.85, 56.38, 56.27, 47.21, 42.05, 25.68. HRMS (ESI) calculated for C_32_H_31_BrNO_4_^+^ [M + H]^+^ 572.1431, found 572.1432.

#### 3.2.10. 1-(5-(Benzoxy)-2-bromo-4-methoxybenzyl)-6-(benzyloxy)-1,2,3,4-tetrahydro-7-methoxyisoquinoline (**12**)

NaBH_4_ (0.57 g, 15.0 mmol, 1.5 equiv.) was added slowly to a stirred ice-cooled solution of 1-(5-(benzyloxy)-2-bromo-4-methoxybenzyl)-6-(benzyloxy)-3,4-dihydro-7-methoxyisoquinoline (**11**; 5.72 g, 10.0 mmol, 1.0 equiv.) in EtOH (30 mL) at 0 °C. The mixture was stirred at 0 °C for 1 h and then at 20–30 °C for 2 h. The mixture was cooled to 0 °C and diluted with water (10 mL), after which the precipitate was filtered. The precipitate was washed with water to neutral, which furnished 1-(5-(benzyloxy)-2-bromo-4-methoxybenzyl)-6-(benzyloxy)-1,2,3,4-tetrahydro-7-methoxyisoquinoline (**12**) (72% yield).

1-(5-(Benzyloxy)-2-bromo-4-methoxybenzyl)-6-(benzyloxy)-1,2,3,4-tetrahydro-7-methoxyisoquinoline (**12**): white solid; Rf (CH_2_Cl_2_/MeOH 90:10) = 0.55; purification by flash column chromatography (deactivated silica gel, CH_2_Cl_2_) ^1^H-NMR (400 MHz, DMSO-*d*_6_): *δ* = 9.16 (br, 1H), 7.41–7.32 (m, 10H), 7.28 (s, 1H), 7.18 (s, 1H), 6.88 (s, 1H), 6.36 (s, 1H), 5.07 (d, *J* = 11.6 Hz, 1H), 5.03 (s, 2H), 4.98 (d, *J* = 11.6 Hz, 1H), 4.53 (t, *J* = 7.4 Hz, 2H), 3.75 (s, 3H), 3.53 (s, 3H), 3.41–3.33 (m, 2H), 3.20–3.15 (m, 2H), 3.02–2.95 (m, 1H), 2.87–2.79 (m, 1H); ^13^C-NMR (100 MHz, DMSO-*d*_6_): *δ* = 149.59, 147.90, 147.80, 137.47, 137.13, 130.19, 129.00 (2C), 128.96 (2C), 128.58 (2C), 128.43, 128.31 (2C), 127.74, 125.02, 117.68, 116.44, 115.43, 113.93, 110.55, 70.74, 70.34, 56.57, 55.88, 54.35, 39.19, 29.61, 29.12, 27.08, 25.37. HRMS (ESI) data were calculated for C_32_H_33_BrNO_4_^+^ [M + H]^+^ 574.1588, found 574.1587.

#### 3.2.11. Tert-Butyl 1-(5-(benzyloxy)-2-bromo-4-methoxybenzyl)-6-(benzyloxy)-3,4-dihydro-7-methoxyisoquinoline-2(1H)-carboxylate (**13**)

Triethylamine (1.32 g, 13.0 mmol, 1.3 equiv.) and Boc_2_O (2.62 g, 12.0 mmol, 1.2 equiv.) were added to a stirred solution of 1-(5-(benzyloxy)-2-bromo-4-methoxybenzyl)-6-(benzyloxy)-1,2,3,4-tetrahydro-7-methoxyisoquinoline (**12**; 5.75 g, 10.0 mmol, 1 equiv.) in CH_2_Cl_2_ (15 mL) at 20–30 °C, and the resulting mixture was stirred for 2 h and washed sequentially with water (2 × 10 mL) and brine (2 × 10 mL). The organic layer was dried (anhydrous NaSO_4_) and concentrated under reduced pressure, which furnished tert-butyl1-(5-(benzyloxy)-2-bromo-4-methoxybenzyl)-6-(benzyloxy)-3,4-dihydro-7-methoxyisoquinoline-2(1H)-carboxylate (**13**) (80% yield).

Tert-butyl1-(5-(benzyloxy)-2-bromo-4-methoxybenzyl)-6-(benzyloxy)-3,4-dihydro-7-methoxyisoquinoline-2(1H)-carboxylate (**13**): white solid; Rf (CH_2_Cl_2_/MeOH 97:3) = 0.52; purification by flash column chromatography (deactivated silica gel, CH_2_Cl_2_) ^1^H-NMR (400 MHz, CDCl_3_): *δ* = 7.44–7.27 (m, 10H), 7.06 (s, 1H), 6.72 (s, 1H), 6.63 (s, 1H), 6.55 (s, 1H), 5.24 (dd, *J* = 10.1, 3.8 Hz, 1H), 5.11–5.05 (m, 4H), 4.27–4.21 (m, 1H), 3.86 (s, 3H), 3.84 (s, 3H), 3.18–3.13 (m, 2H), 2.85–2.79 (m, 2H), 2.57–2.52 (m, 1H), 1.20 (s, 9H); ^13^C-NMR (400 MHz, CDCl_3_): *δ* = 154.17, 149.84, 146.58, 143.65, 130.39, 130.28, 127.43, 123.82, 123.01, 115.68, 114.98, 112.55, 79.48, 67.53, 59.61, 56.34, 56.10, 30.02, 28.63 (3C), 28.22, 25.64. HRMS (ESI) calculated for C_37_H_41_BrNO_6_^+^ [M + H]^+^ 674.2112, found 674.2114.

#### 3.2.12. Tert-Butyl 1-(5-(Benzyloxy)-2-bromo-4-methoxybenzyl)-6-(benzyloxy)-3,4-dihydro-7-methoxyisoquinoline-2(1H)-carboxylate (**14**)

Pd(OAc)_2_ (4.49 g, 2.0 mmol, 0.2 equiv.), the ligand di-tert-butyl(methyl)phosphonium tetrafluoroborate (9.89 g, 4.0 mmol, 0.4 equiv.), and Cs_2_CO_3_ (9.77 g, 3.0 mmol, 3 equiv.) were added to a solution of tert-butyl1-(5-(benzyloxy)-2-bromo-4-methoxybenzyl)-6-(benzyloxy)-3,4-dihydro-7-methoxyisoquinoline-2(1H)-carboxylate (**13**; 6.75 g, 10.0 mmol, 1 equiv.) in 1,4-dioxane (40 mL) by purging with nitrogen for 8 h at 101 °C. After cooling to 20–30 °C, the solvent was removed under reduced pressure. The precipitate was extracted with CH_2_Cl_2_ (3 × 50 mL). The combined organic layer was dried (anhydrous Na_2_SO_4_), and the precipitate was loaded onto a deactivated silica gel column (200–300 mesh) and eluted with hexane/EtOAc (90:10, *v*/*v*) to afford 6H-dibenzo[de,g]quinoline-6-carboxylic acid, 4,5,6a,7-tetrahydro-1,9,10-trimethoxy-2-(phenylmethoxy)-,1,1-dimethylethyl ester (**14**) (53%).

6H-Dibenzo[de,g]quinoline-6-carboxylic acid, 4,5,6a,7-Tetrahydro-1,9,10-trimethoxy-2-(phenylmethoxy)-, 1,1-dimethylethyl ester (**14**): white solid; Rf (Hexanes/EtOAc 80:20) = 0.64; purification by flash column chromatography (deactivated silica gel, Hexanes/EtOAc 94:6) ^1^H-NMR (400 MHz, CDCl_3_): *δ* = 8.17 (s, 1H), 7.47–7.27 (m, 10H), 6.77 (s, 1H), 6.67 (s, 1H), 5.19–5.08 (m, 4H), 4.63–4.60 (m, 1H), 4.38–4.35 (m, 1H), 3.91 (s, 3H), 3.70 (s, 3H), 2.92–2.68 (m, 5H), 2.59 (d, *J* = 14.7 Hz, 1H), 1.43 (s, 9H); ^13^C-NMR (400 MHz, CDCl_3_): *δ* = 154.28, 149.23, 148.14, 147.81, 147.19, 137.18, 136.96, 130.35, 129.53, 128.75 (2C), 128.66 (2C), 128.05, 127.96, 127.37 (2C), 127.33 (2C), 126.71, 117.20, 116.02, 115.71, 114.15, 110.71, 79.47, 71.68, 71.13, 56.55, 56.27, 54.33, 42.12, 36.48, 28.53, 28.17 (3C). HRMS (ESI) calculated for C_37_H_39_NO_6_Na^+^ [M + Na]^+^ 616.2670, found 616.2667.

#### 3.2.13. Tert-Butyl 6H-Dibenzo[de,g]quinoline-6-carboxylic acid,4,5,6a,7-tetrahydro-9-hydroxy-1,2,10-trimethoxy-,1,1-dimethylethyl Ester (**15**)

Palladium 10% on carbon (wetted with ca. 55% water) (Pd/C) (1.18 g) and HOAc (0.5 mL) were added to a solution of 6H-dibenzo[de,g]quinoline-6-carboxylic acid, 4,5,6a,7-tetrahydro-1,9,10-trimethoxy-2-(phenylmethoxy)-, and 1,1-dimethylethyl ester (**14**; 5.94 g, 10.0 mmol, 1 equiv.) in THF (40 mL) by purging with hydrogen for 8 h at 20–30 °C. The precipitate was filtered off. The solvent was removed under reduced pressure, and the obtained residue was dissolved in EtOAc (40 mL) and washed sequentially with water (2 × 20 mL) and brine (2 × 20 mL). The organic layer was dried (anhydrous NaSO_4_) and concentrated under reduced pressure, which furnished 6H-dibenzo[de,g]quinoline-6-carboxylic acid and 4,5,6a,7-tetrahydro-9-hydroxy-1,2,10-trimethoxy-, 1,1-dimethylethyl ester (**15**) (82% yield).

6H-Dibenzo[de,g]quinoline-6-carboxylic acid,4,5,6a,7-tetrahydro-9-hydroxy-1,2,10-trimethoxy-,1,1-dimethylethyl ester (**15**): white solid; Rf (Hexanes/EtOAc 66:34) = 0.46; purification by flash column chromatography (deactivated silica gel, Hexanes/EtOAc 80:20) ^1^H-NMR (400 MHz, DMSO-*d*_6_): *δ* = 9.18 (s, 1H), 9.16 (s, 1H), 7.89 (s, 1H), 6.65 (s, 1H), 6.54 (s, 1H), 4.38–4.33 (m, 1H), 4.15 (d, *J* = 12.7 Hz, 1H), 3.75 (s, 3H), 3.51 (s, 3H), 2.66–2.49 (m, 5H), 1.37 (s, 9H); ^13^C-NMR (100 MHz, DMSO-*d*_6_): *δ* = 154.17, 149.84, 146.58, 143.65, 130.39, 130.28, 127.43, 123.82, 123.01, 115.68, 114.98, 112.55, 79.48, 67.53, 59.61, 56.34, 56.10, 30.02, 28.63 (3C), 28.22, 25.64. HRMS (ESI) calculated for C_23_H_27_NO_6_Na^+^ [M + H]^+^ 436.1731, found 436.1729.

#### 3.2.14. 5,6,6a,7-Tetrahydro-1,10-dimethoxy-4H-dibenzo[de,g]quinoline-2,9-diol (**16**)

ZnBr_2_ (9.01 g, 40.0 mmol, 4 equiv.) was added to a solution of 6H-dibenzo[de,g]quinoline-6-carboxylic acid, 4,5,6a,7-tetrahydro-9-hydroxy-1,2,10-trimethoxy-, 1,1-dimethylethyl ester (**15**; 4.13 g, 10.0 mmol, 1 equiv.) in dry CH_2_Cl_2_ (50 mL) under a nitrogen atmosphere, and the mixture was stirred at 20–30 °C for 48 h. The mixture was then quenched with a solution of saturated NaHCO_3_ (50 mL) and extracted with CH_2_Cl_2_ (3 × 250 mL). The combined organic layer was dried (anhydrous Na_2_SO_4_) and concentrated under reduced pressure to afford the resultant crude products. Then, the resultant crude products were purified by column chromatography on silica gel (CH_2_Cl_2_/MeOH 90:10), which furnished 5,6,6a,7-Tetrahydro-1,10-dimethoxy-4H-dibenzo[de,g]quinoline-2,9-diol (**16**) (49% yield).

5,6,6a,7-Tetrahydro-1,10-dimethoxy-4H-dibenzo[de,g]quinoline-2,9-diol (**16**): brown solid; Rf (CH_2_Cl_2_/MeOH 80:20) = 0.45; purification by flash column chromatography (deactivated silica gel, CH_2_Cl_2_/MeOH 90:10) ^1^H-NMR (400 MHz, DMSO-*d*_6_): *δ* = 10.21 (br, 1H), 9.48 (s, 1H), 9.37 (s, 1H), 7.85 (s, 1H), 6.73 (s, 1H), 6.62 (s, 1H), 4.09–4.06 (m, 1H), 3.73 (s, 3H), 3.55 (s, 3H), 3.46–3.44 (m, 1H), 3.13–3.02 (m, 2H), 2.89–2.84 (m, 1H), 2.81–2.73 (m, 2H); ^13^C-NMR (100 MHz, DMSO-*d*_6_): *δ* = 151.23, 147.01, 146.88, 143.85, 127.11, 127.07, 126.78, 122.73, 120.16, 115.75, 114.94, 112.54, 59.87, 56.16, 52.42, 40.61, 32.82, 25.14. HRMS (ESI) calculated for C_18_H_20_NO_4_^+^ [M + H]^+^ 314.1387, found 314.1384.

## 4. Conclusions

The total synthesis of laurolitsine, an alkaloid extracted from *Litsea glutinosa* bark, was achieved in 14 steps with a 2.3% yield (this was calculated using 3-hydroxy-4-methoxybenzaldehyde as the starting material) starting from 3-hydroxy-4-methoxybenzaldehyde (**1**) and 2-(3-hydroxy-4-methoxyphenyl)acetic acid (**5**), and the longest linear sequence consisted of 11 steps. In this study, many experimental steps were optimized, and an innovative postprocessing method for salting 2-(3-(benzyloxy)-4-methoxyphenyl)ethanamine (**4**) with oxalic acid was proposed.

## Figures and Tables

**Table 1:** Comparison of the ^13^C NMR data of **16** with literature data [4].

No.	Natural (*δ*_1_)	Sample 16 (*δ*_2_)	Δ = *δ*_1_ − *δ*_2_
1	143.1	143.9	−0.8
1a	127.1	126.8	0.3
1b	126.3	122.7	3.6
2	149.6	151.2	−1.6
3	115.1	114.9	0.2
3a	129.7	127.1	2.6
4	28.9	25.1	3.8
5	43.1	40.6	2.5
6a	53.9	52.4	1.5
7	36.5	32.8	3.7
7a	130.1	127.1	3.0
8	115.6	115.8	−0.2
9	146.5	147.0	−0.5
10	146.3	146.9	−0.6
11	112.6	112.5	0.1
11a	123.5	120.2	3.3
1-OCH_3_	59.7	59.9	−0.2
10-OCH_3_	56.2	56.2	0

## Data Availability

Data are contained within the article.

## Supplementary Materials

The following supporting information can be downloaded at https://www.mdpi.com/article/10.3390/molecules29030745/s1. Detailed procedures for the synthesis of **16** and ^1^H- and ^13^C-NMR charts of all the compounds.
