# Supplementary material for: Total Synthesis of the Racemate of Laurolitsine
Source: Molecules. 2024 Feb 5;29(3):745. doi: 10.3390/molecules29030745 (PMC10856274; doi:10.3390/molecules29030745)
Supplement: Supplementary file 1 [file molecules-29-00745-s001.zip › molecules-2848445-supplementary.pdf]

molecules

MDPI

Supporting Information for

Total Synthesis of the Racemate of Laurolitsine

Mingyu Cao [1], Yiming Wang [1], Yong Zhang[1], Caiyun Zhang[1], Niangen Chen[1,*] and Xiaopo Zhang[1,2,*]

1 Key Laboratory of Tropical Translational Medicine of Ministry of Education, Hainan Key Laboratory for Research and Development of Tropical Herbs, School of Pharmacy, Hainan Medical University, Haikou 571199, China
2 Research Center for Drug Safety Evaluation of Hainan Province, Hainan Medical University, Haikou 571101, China
3 Correspondence: hy0207011@hainmc.edu.cn (N. C.); hy0207077@hainmc.edu.cn (X. Z.). Tel: +86-0898-66893826 (X. Z.)

Table of Contents

- ^{1}H NMR and ^{13}C NMR spectra of **2**……S2-S3
- ^{1}H NMR and ^{13}C NMR spectra of **3**……S4-S5
- ^{1}H NMR and ^{13}C NMR spectra of **4**……S6-S7
- ^{1}H NMR and ^{13}C NMR spectra of **6**……S8-S9
- ^{1}H NMR and ^{13}C NMR spectra of **7**……S10-S11
- ^{1}H NMR and ^{13}C NMR spectra of **8**……S12-S13
- ^{1}H NMR and ^{13}C NMR spectra of **9**……S14-S15
- ^{1}H NMR and ^{13}C NMR spectra of **10**……S16-S17
- ^{1}H NMR and ^{13}C NMR spectra of **11**……S18-S19
- ^{1}H NMR and ^{13}C NMR spectra of **12**……S20-S21
- ^{1}H NMR and ^{13}C NMR spectra of **13**……S22-S23
- ^{1}H NMR and ^{13}C NMR spectra of **14**……S24-S25
- ^{1}H NMR and ^{13}C NMR spectra of **15**……S26-S27
- ^{1}H NMR and ^{13}C NMR spectra of Laurolitsine (**16**)……S28-S29

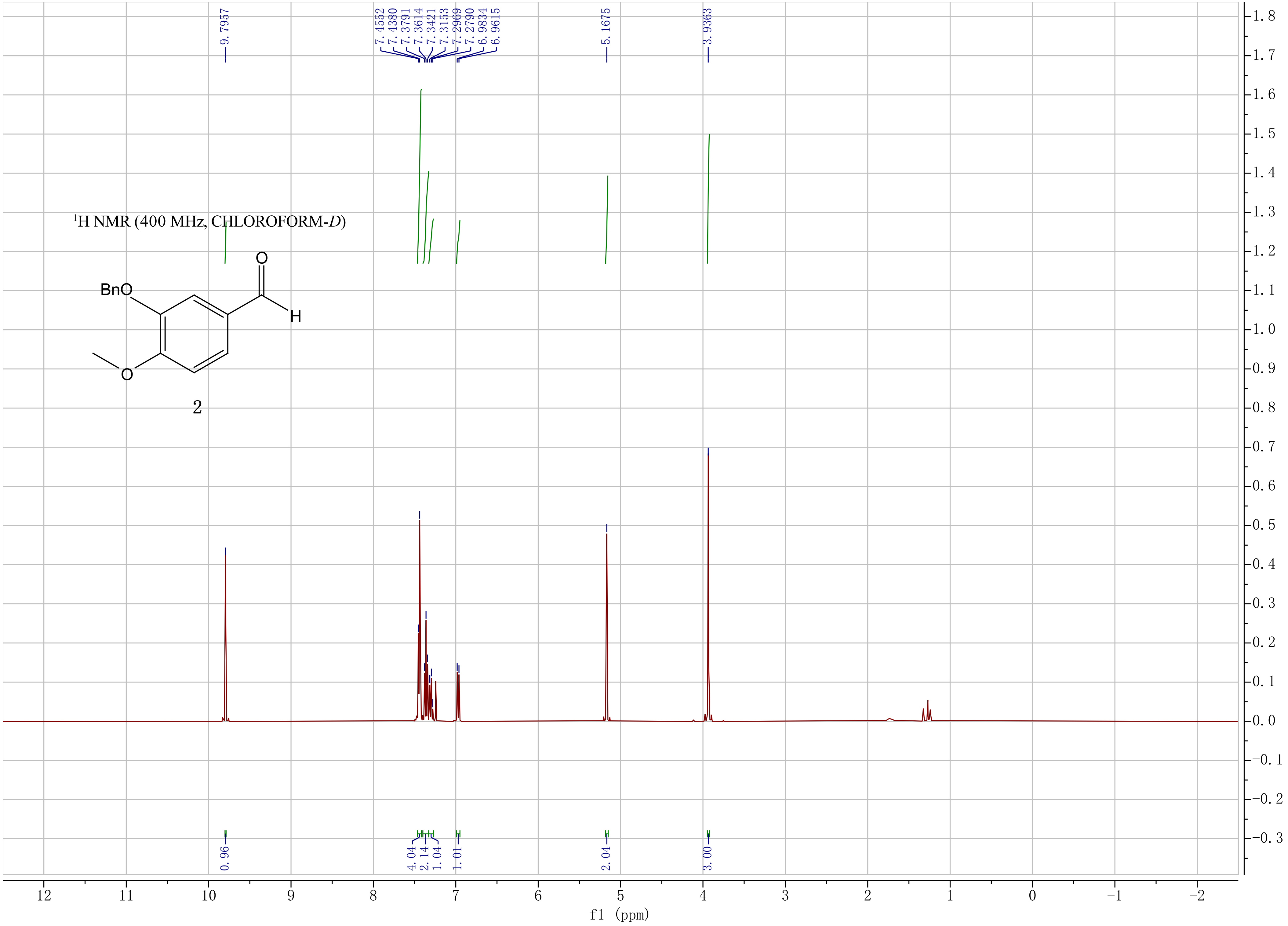
9.7957
7.4552
7.4380
7.3791
7.3614
7.3421
7.3153
7.2969
7.2790
6.9834
6.9615
5.1675
3.9363
^{1}H NMR (400 MHz, CHLOROFORM-D)
O
BnO
H
O
2
0.96
4.04
2.14
1.04
1.01
2.04
3.00
f1 (ppm)

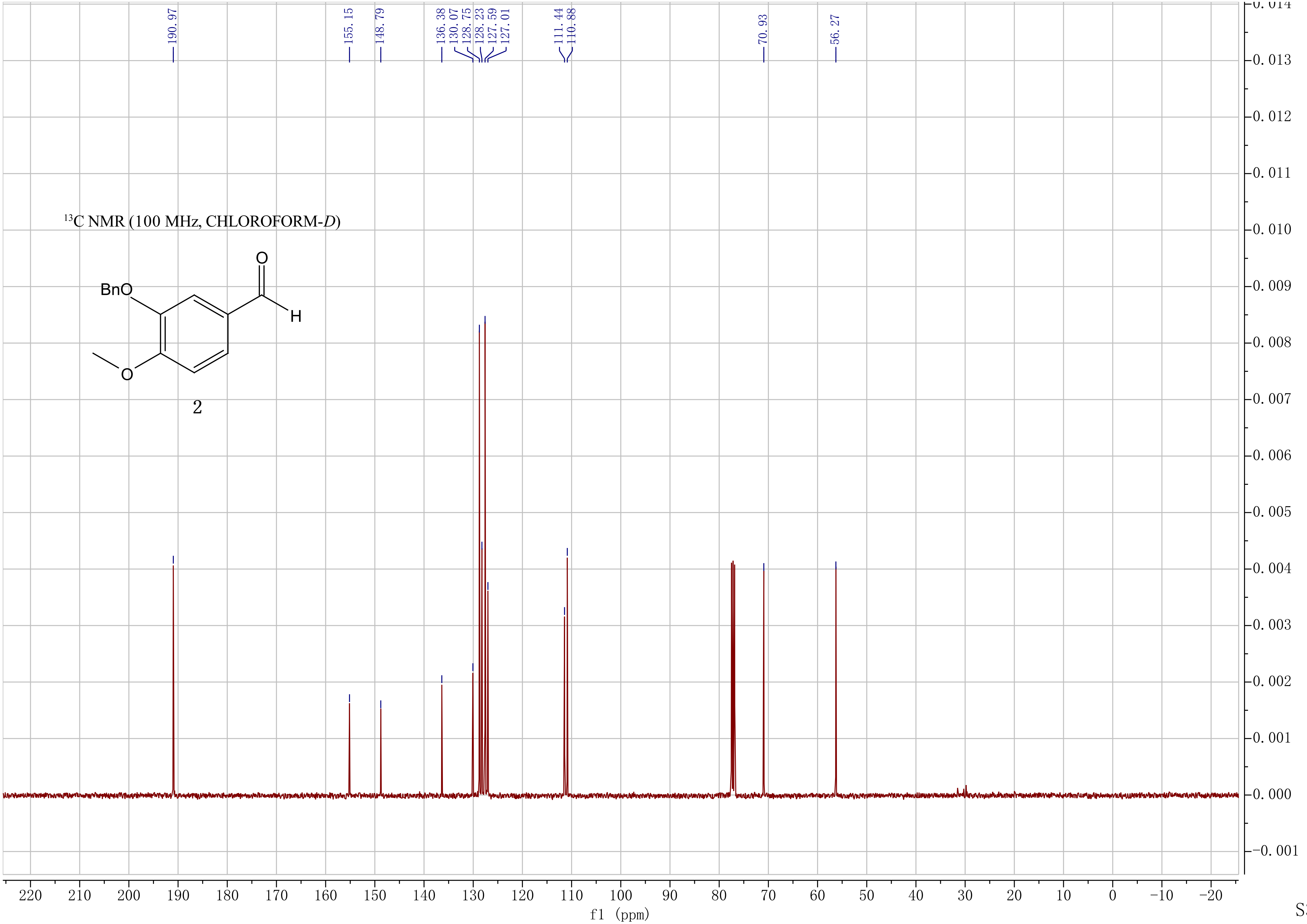
190.97
155.15
148.79
136.38
130.07
128.75
128.23
127.59
127.01
111.44
110.88
70.93
56.27
13C NMR (100 MHz, CHLOROFORM-D)
BnO
O
H
O
2
f1 (ppm)

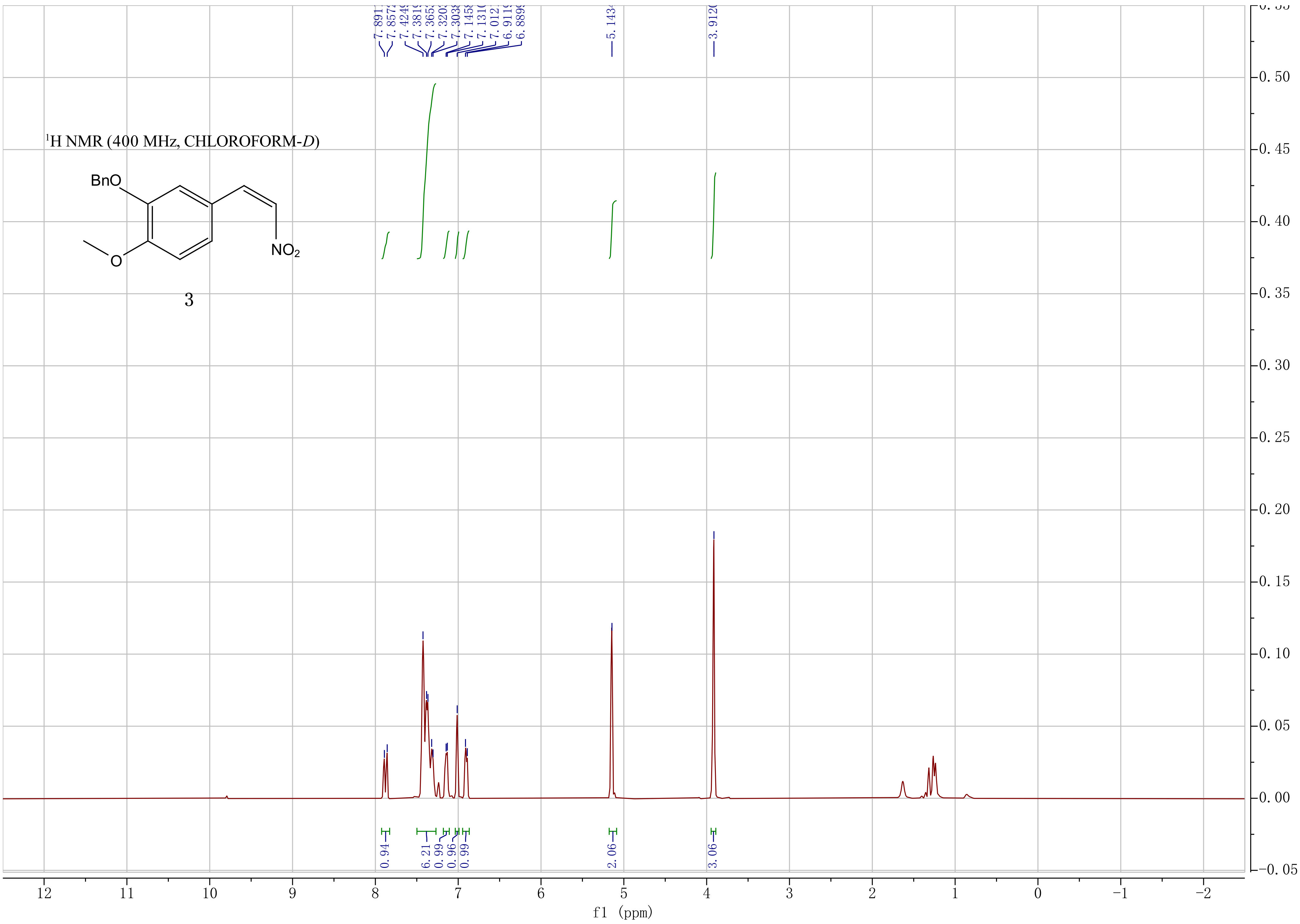

1H NMR (400 MHz, CHLOROFORM-D)
BnO
O
NO2
3
7.4249
0.94
6.21
0.99
0.96
0.99
2.06
3.06
f1 (ppm)

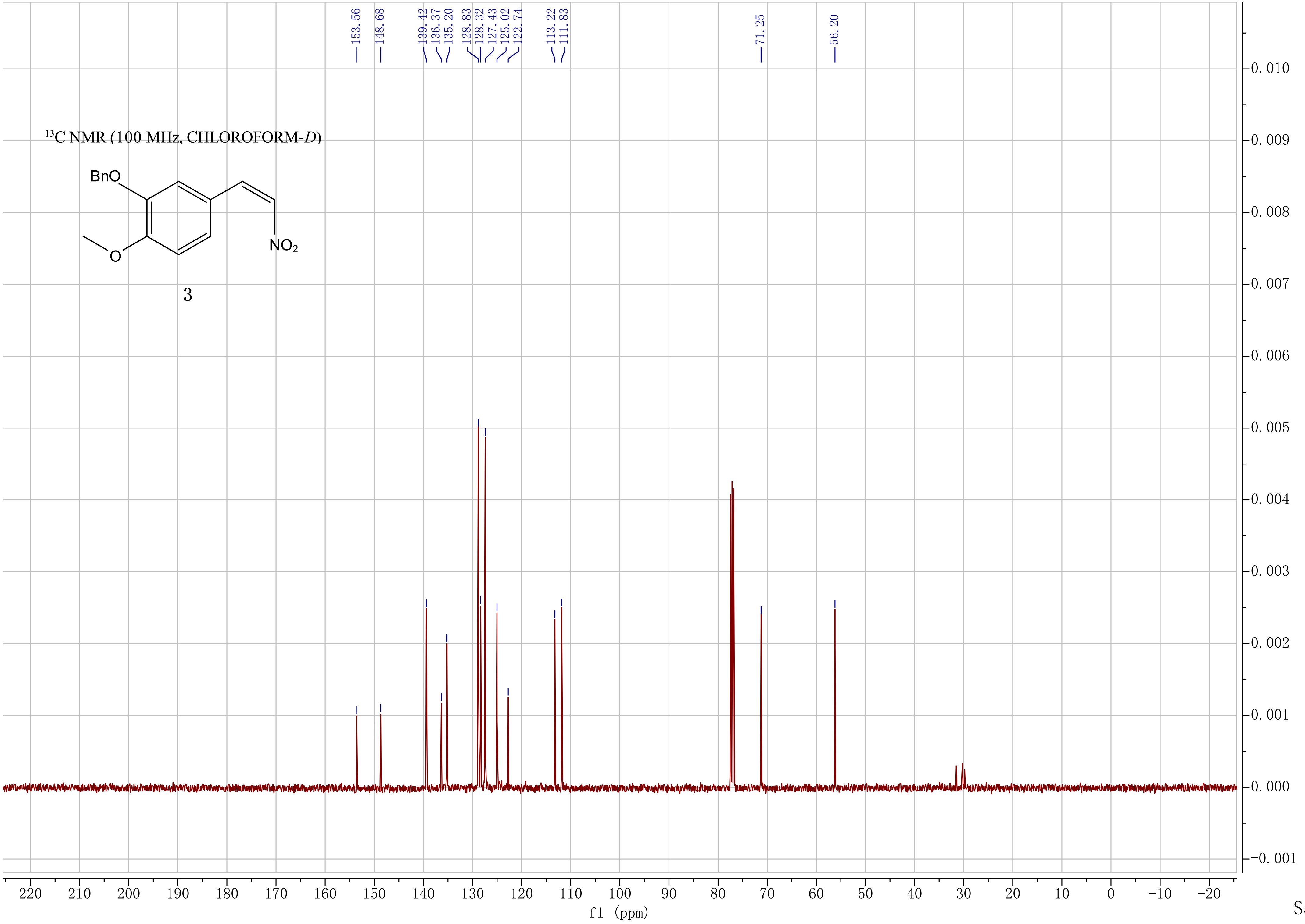
153.56
148.68
139.42
136.37
135.20
128.83
128.32
127.43
125.02
122.74
113.22
111.83
71.25
56.20
13C NMR (100 MHz, CHLOROFORM-D)
BnO
O
NO2
3
f1 (ppm)

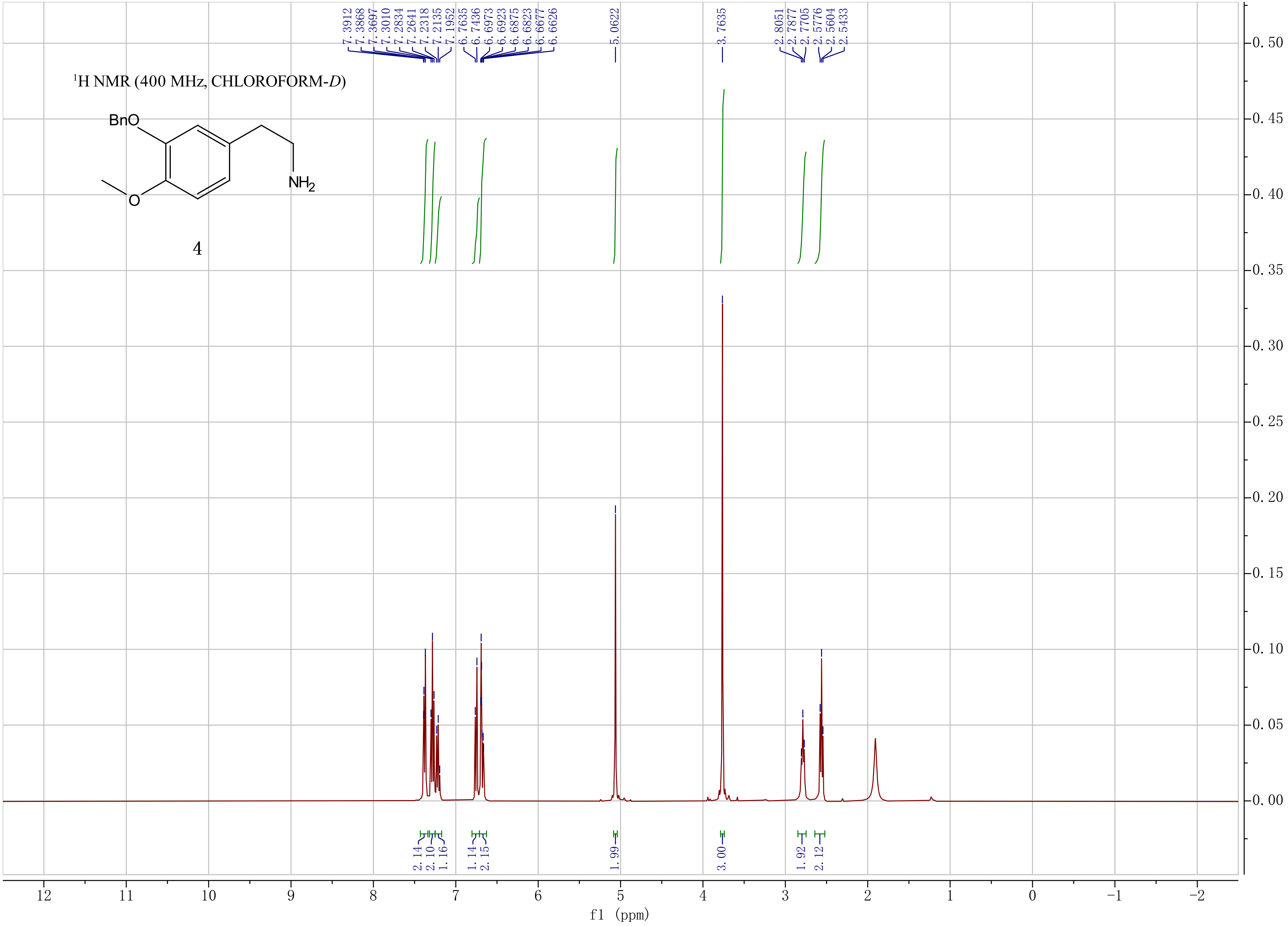
^{1}H NMR (400 MHz, CHLOROFORM-D)
BnO
O
NH2
4
7.3912
7.3868
7.3697
7.3010
7.2834
7.2641
7.2318
7.2135
7.1952
6.7635
6.7436
6.6973
6.6923
6.6875
6.6823
6.6677
6.6626
5.0622
3.7635
2.8051
2.7877
2.7705
2.5776
2.5604
2.5433
2.14
2.10
1.16
1.14
2.15
1.99
3.00
1.92
2.12
f1 (ppm)

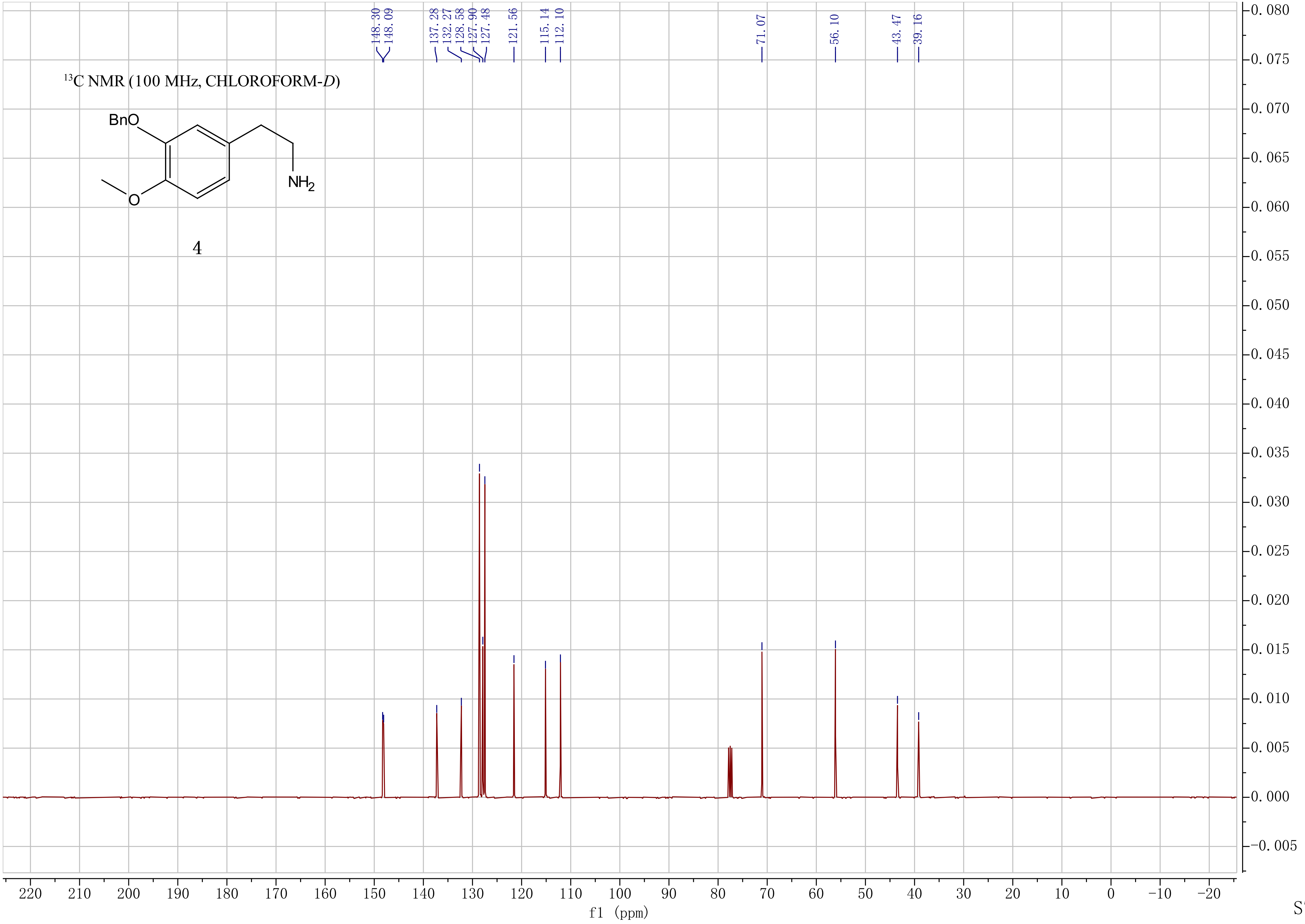

148.30
148.09
137.28
132.27
128.58
127.90
127.48
121.56
115.14
112.10
71.07
56.10
43.47
39.16
13C NMR (100 MHz, CHLOROFORM-D)
BnO
NH2
O
f1 (ppm)

4

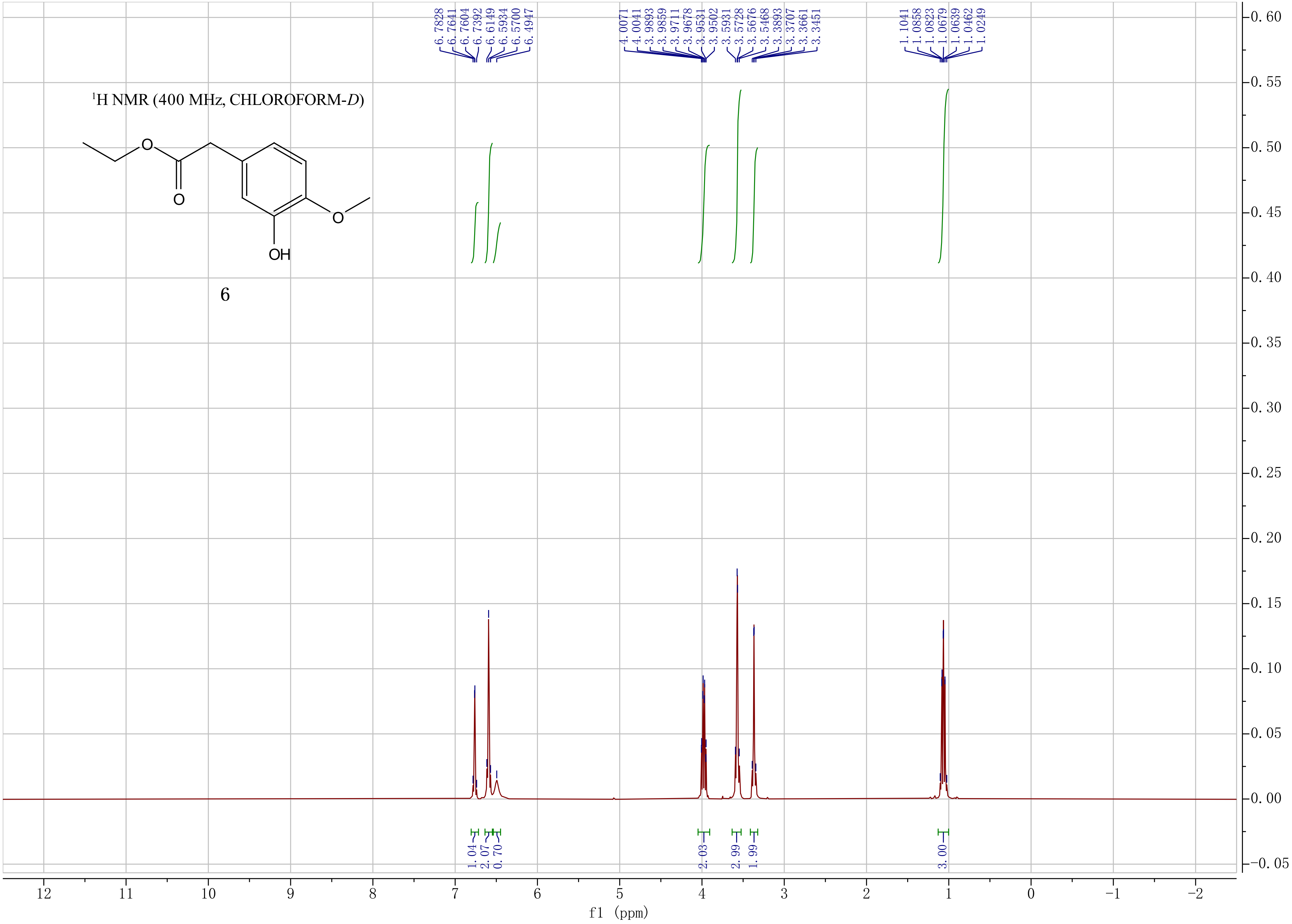

^{1}H NMR (400 MHz, CHLOROFORM-D)
6
OH
6.7828
6.7641
6.7604
6.7392
6.6149
6.5934
6.5700
6.4947
4.0071
4.0041
3.9893
3.9859
3.9711
3.9678
3.9531
3.9502
3.5931
3.5728
3.5676
3.5468
3.3893
3.3707
3.3661
3.3451
1.1041
1.0858
1.0823
1.0679
1.0639
1.0462
1.0249
1.04
2.07
0.70
2.03
2.99
1.99
3.00
f1 (ppm)

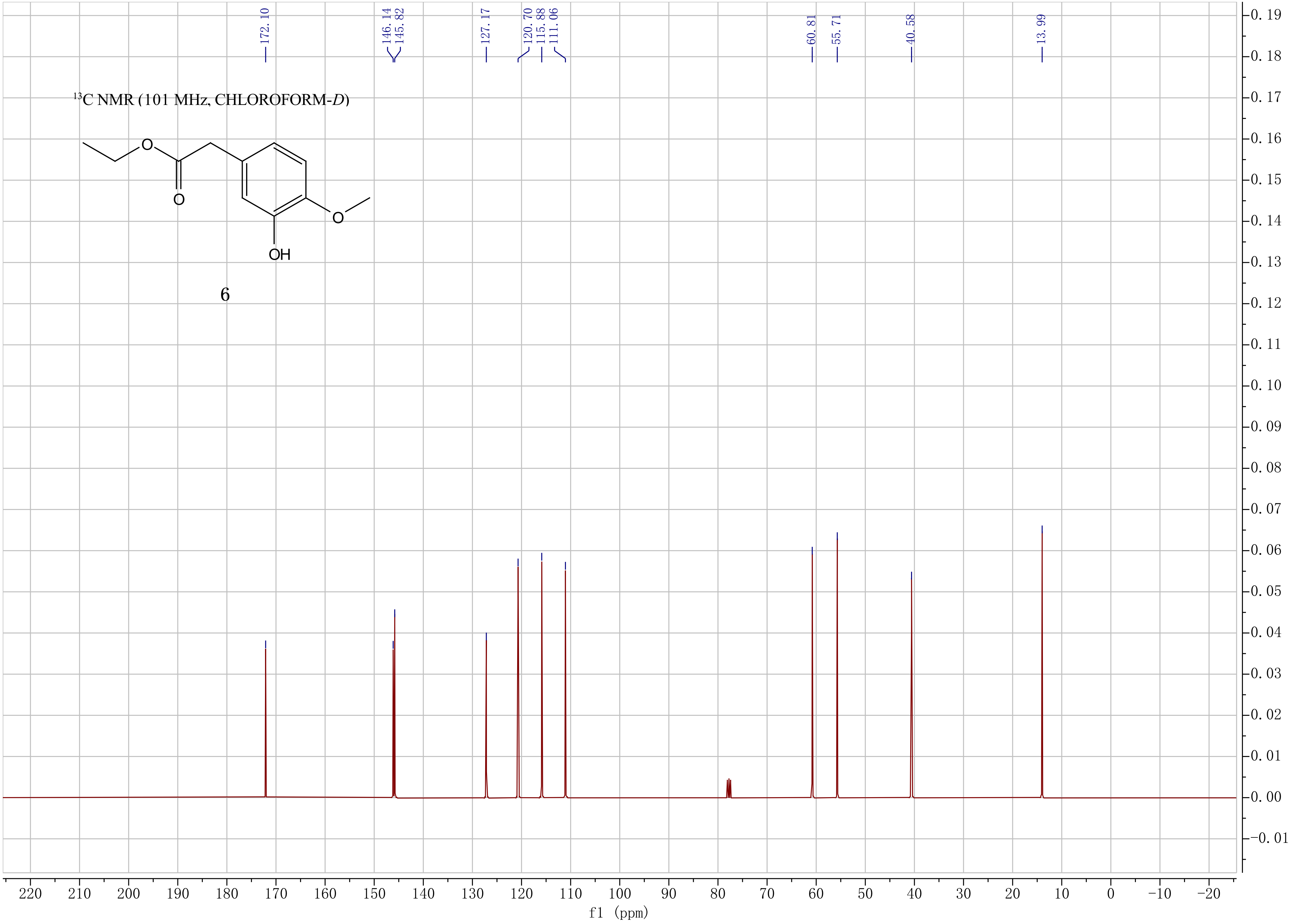
172.10
146.14
145.82
127.17
120.70
115.88
111.06
60.81
55.71
40.58
13.99
13C NMR (101 MHz, CHLOROFORM-D)
O
O
O
OH
6
f1 (ppm)

^{1}H NMR (400 MHz, CHLOROFORM-*D*)

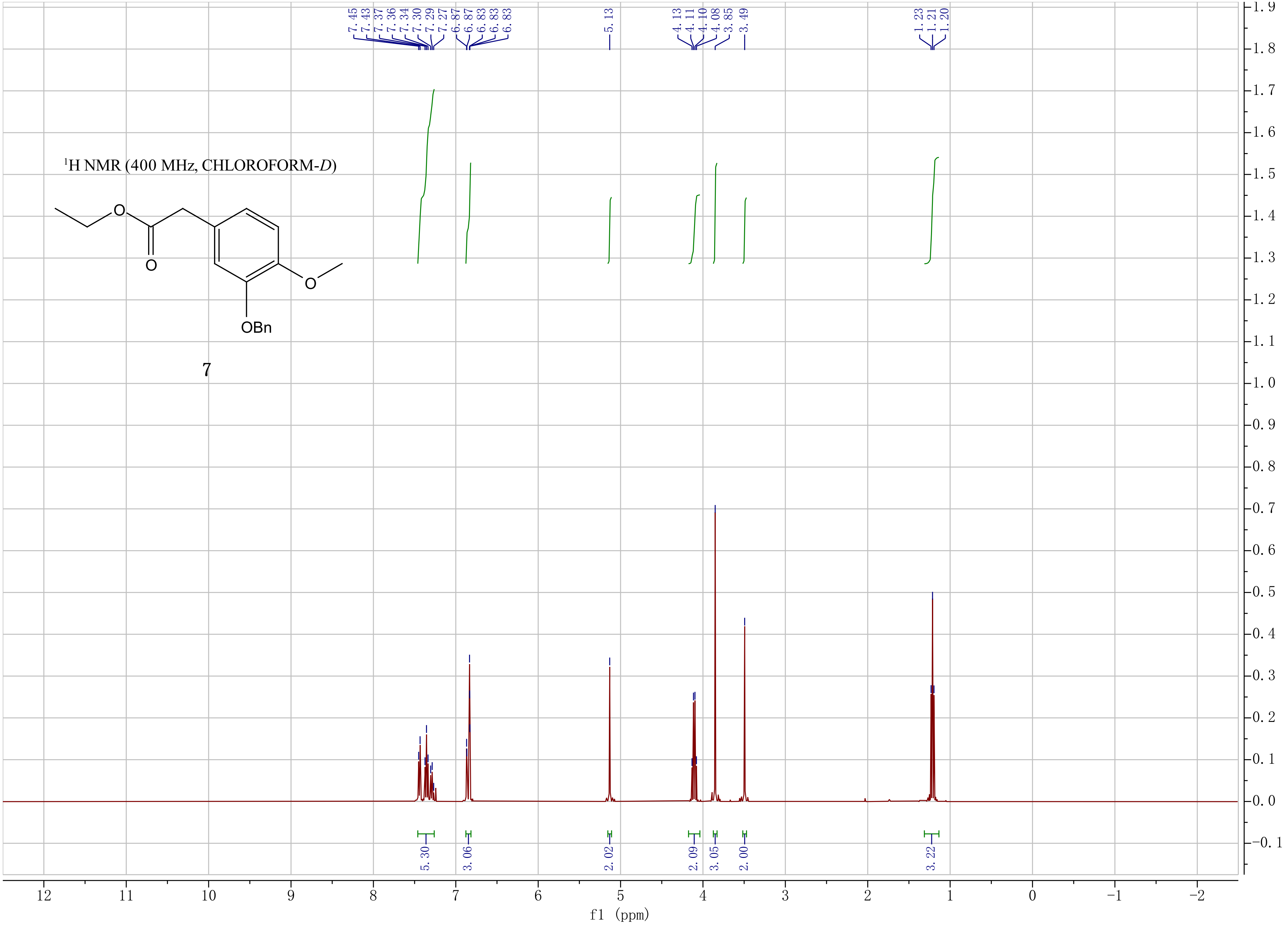

7

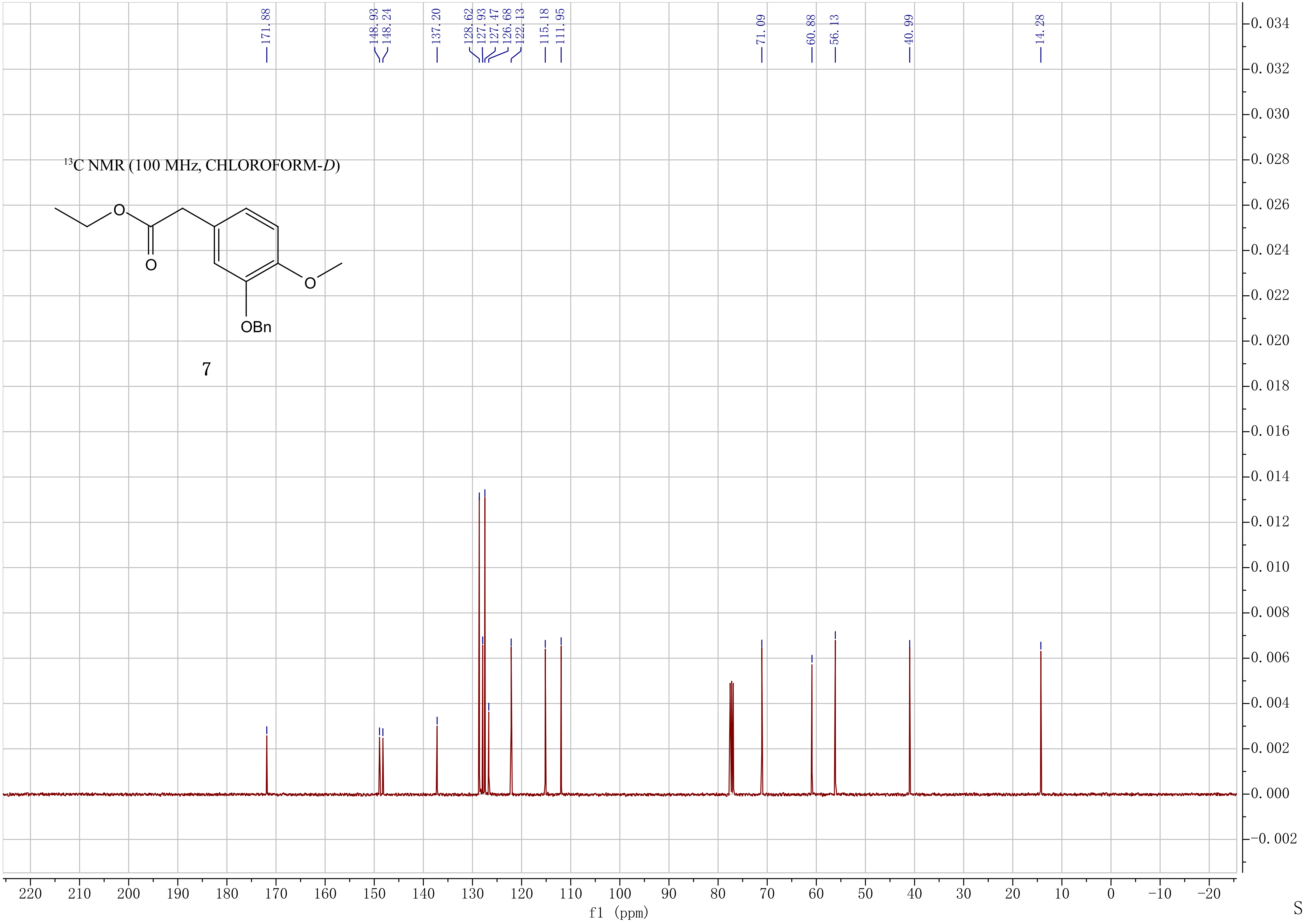

13C NMR (100 MHz, CHLOROFORM-D)
O
O
OBn
7
171.88
148.93
148.24
137.20
128.62
127.93
127.47
126.68
122.13
115.18
111.95
71.09
60.88
56.13
40.99
14.28
f1 (ppm)

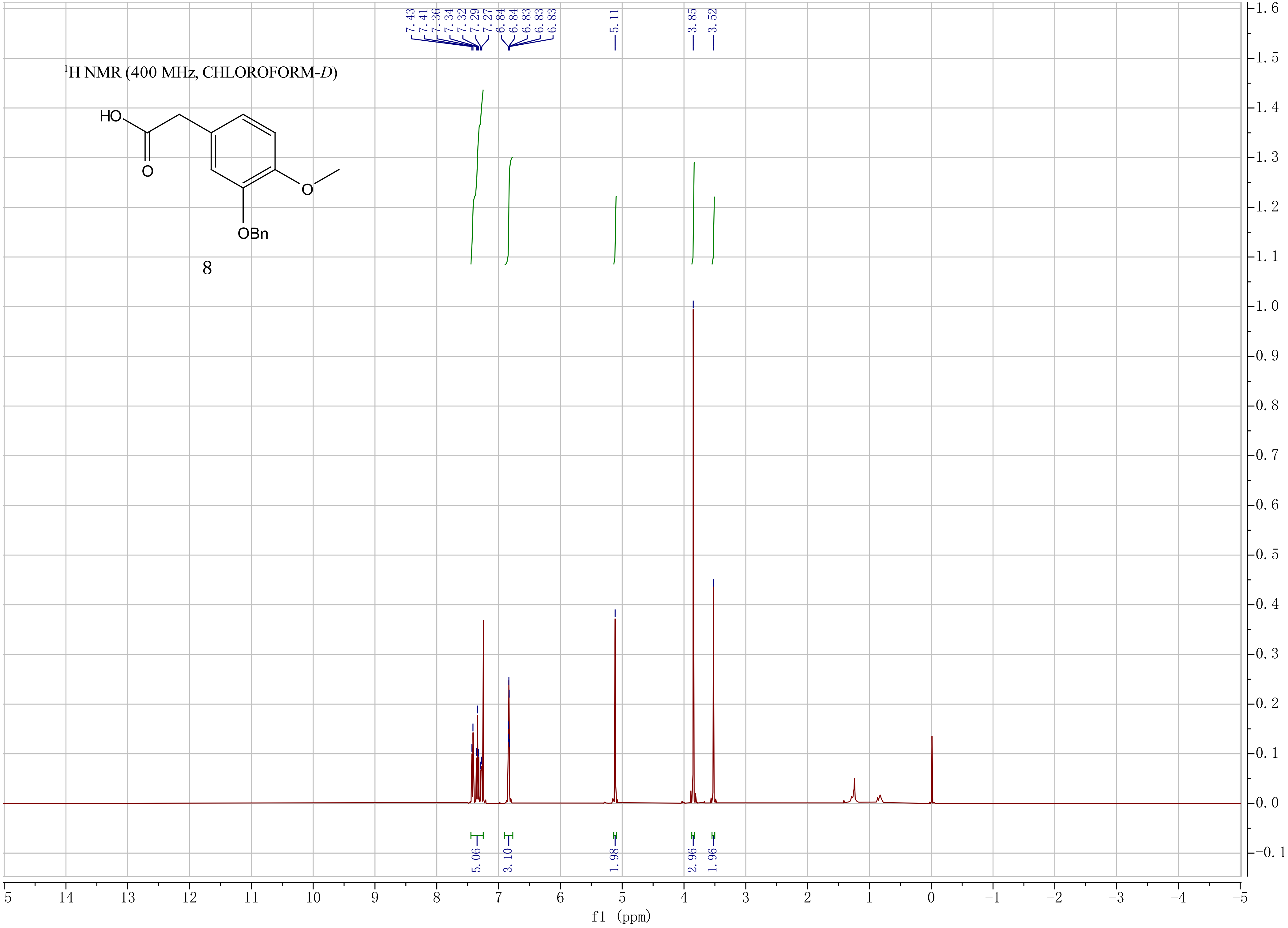

1H NMR (400 MHz, CHLOROFORM-D)
HO
O
O
OBn
8
7.43
7.41
7.36
7.34
7.32
7.29
7.27
6.84
6.84
6.83
6.83
6.83
5.11
3.85
3.52
5.06
3.10
1.98
2.96
1.96
f1 (ppm)

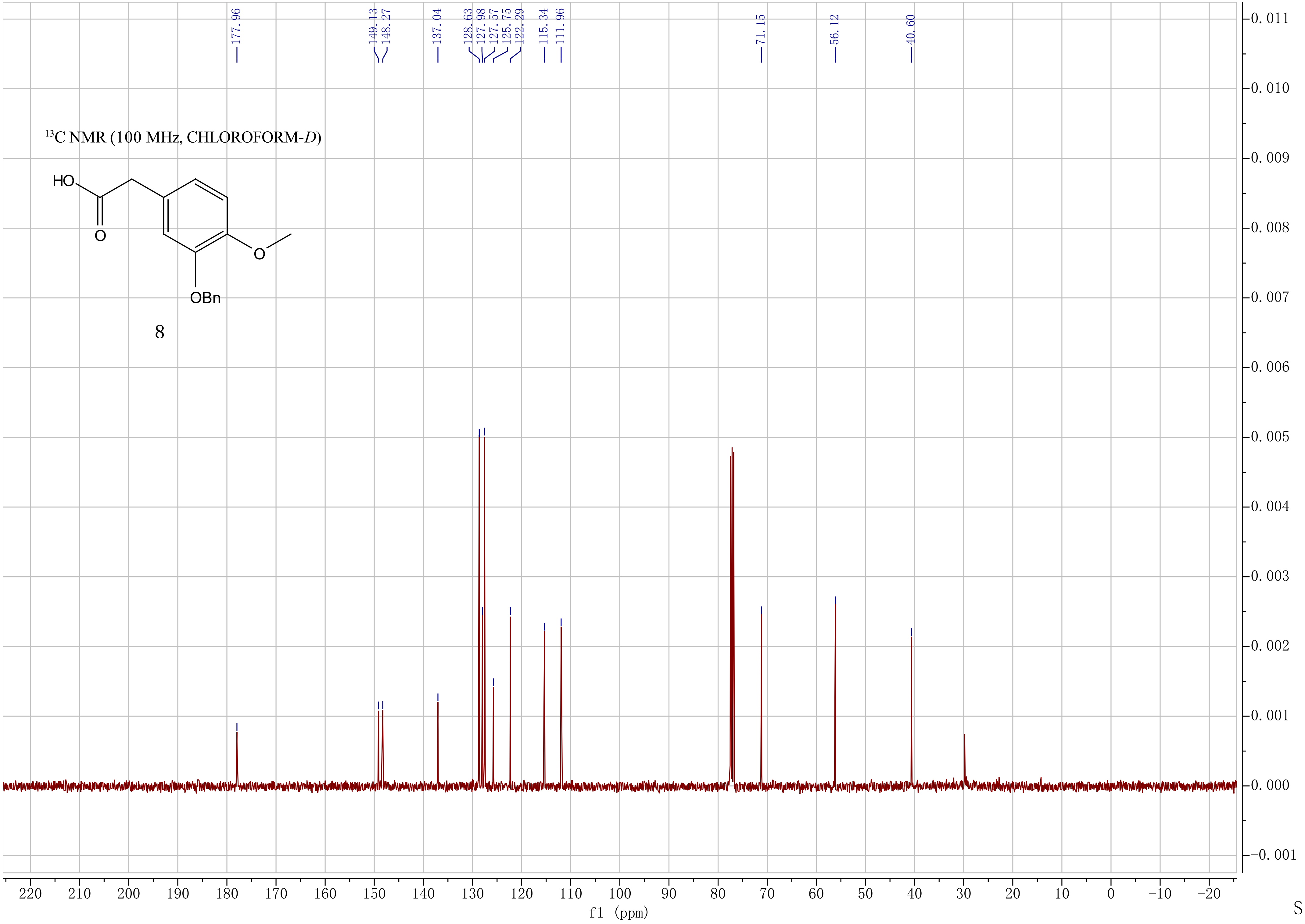

177.96
149.13
148.27
137.04
128.63
127.98
127.57
125.75
122.29
115.34
111.96
71.15
56.12
40.60
¹³C NMR (100 MHz, CHLOROFORM-D)
HO
O
O
OBn
8
f1 (ppm)

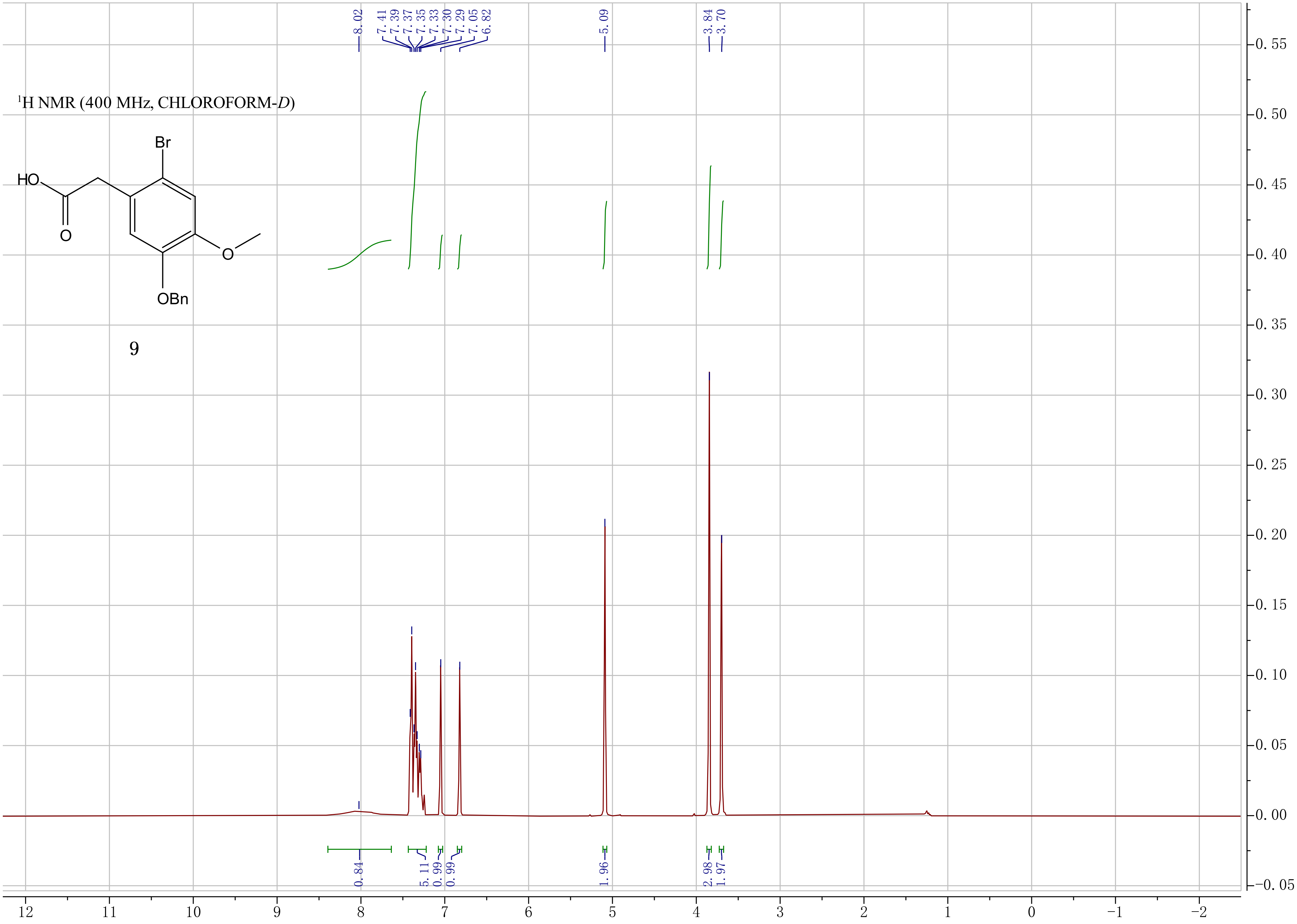
^{1}H NMR (400 MHz, CHLOROFORM-D)
Br
HO
O
O
OBn
9
8.02
7.41
7.39
7.37
7.35
7.33
7.30
7.29
7.05
6.82
5.09
3.84
3.70
0.84
5.11
0.99
0.99
1.96
2.98
1.97

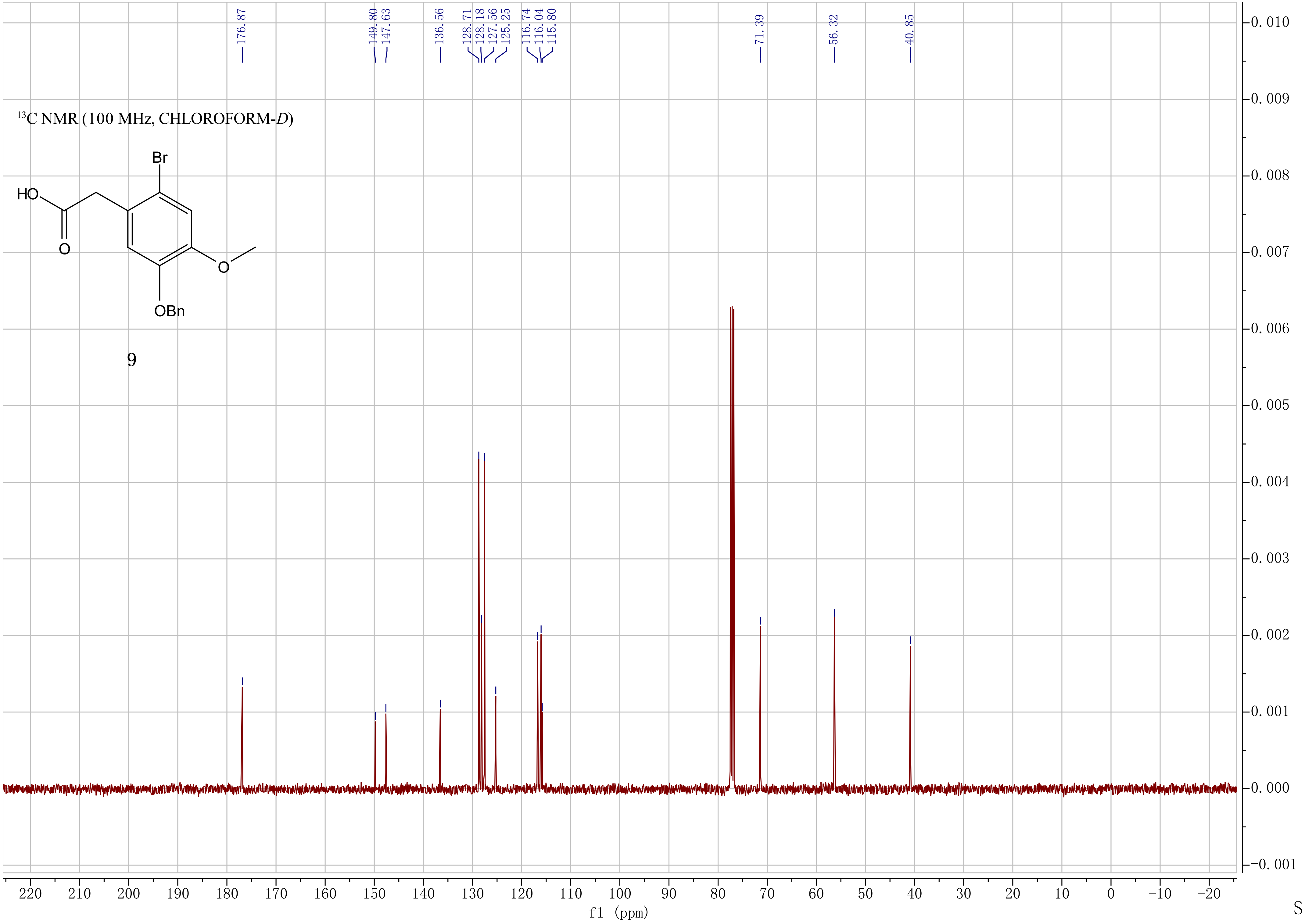

176.87
149.80
147.63
136.56
128.71
128.18
127.56
125.25
116.74
116.04
115.80
71.39
56.32
40.85
13C NMR (100 MHz, CHLOROFORM-D)
Br
HO
O
O
OBn
9
f1 (ppm)

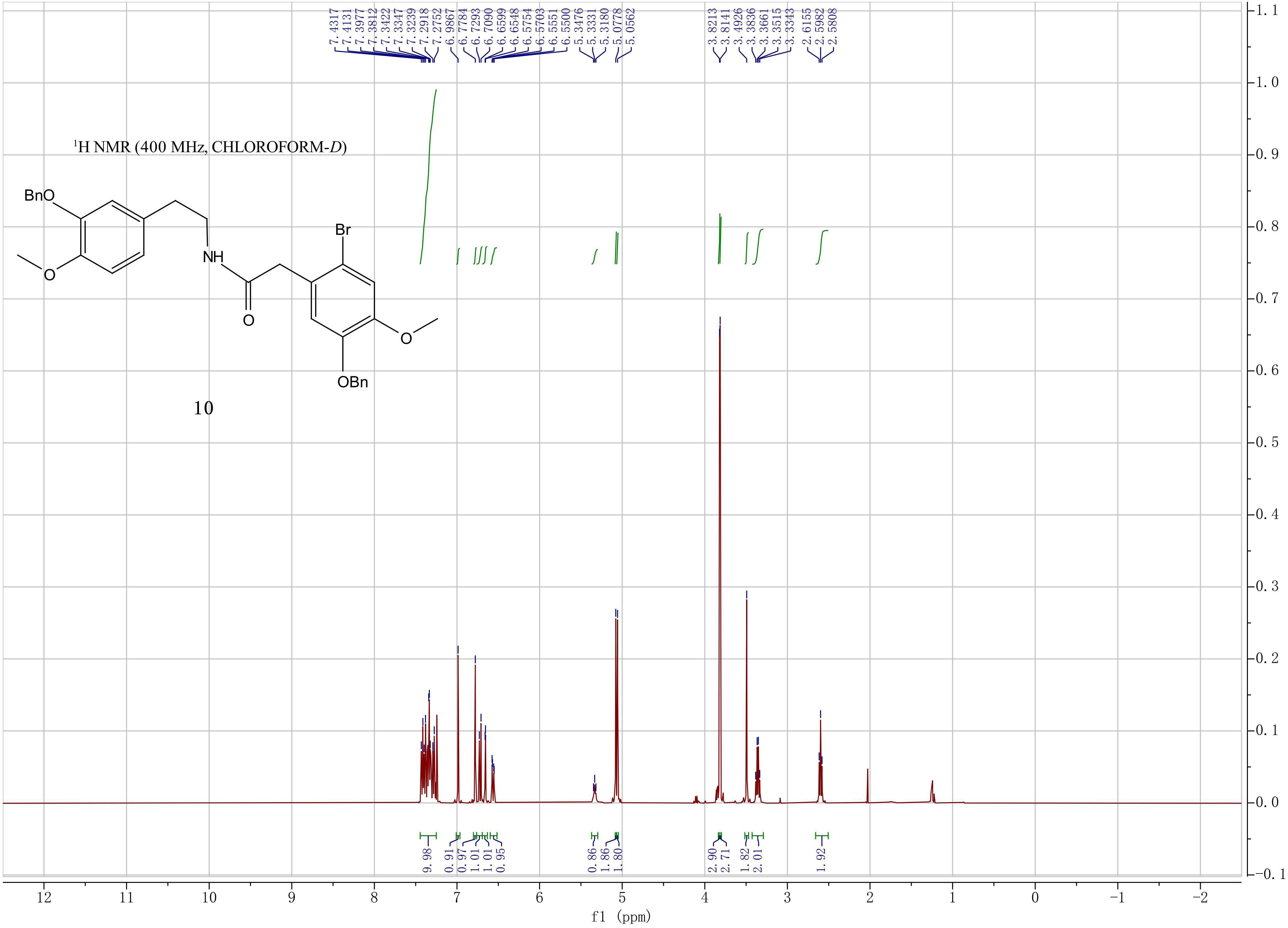

¹H NMR (400 MHz, CHLOROFORM-D)
BnO
NH
Br
O
O
OBn
10
7.4317 7.4131 7.3977 7.3812 7.3422 7.3347 7.3239 7.2918 7.2752 6.9867 6.7784 6.7293 6.7090 6.6599 6.6548 6.5754 6.5703 6.5551 6.5500 5.3476 5.3331 5.3180 5.0778 5.0562
3.8213 3.8141 3.4926 3.3836 3.3661 3.3515 3.3343 2.6155 2.5982 2.5808
9.98 0.91 0.97 1.01 1.01 0.95 0.86 1.86 1.80 2.90 2.71 1.82 2.01 1.92
f1 (ppm)

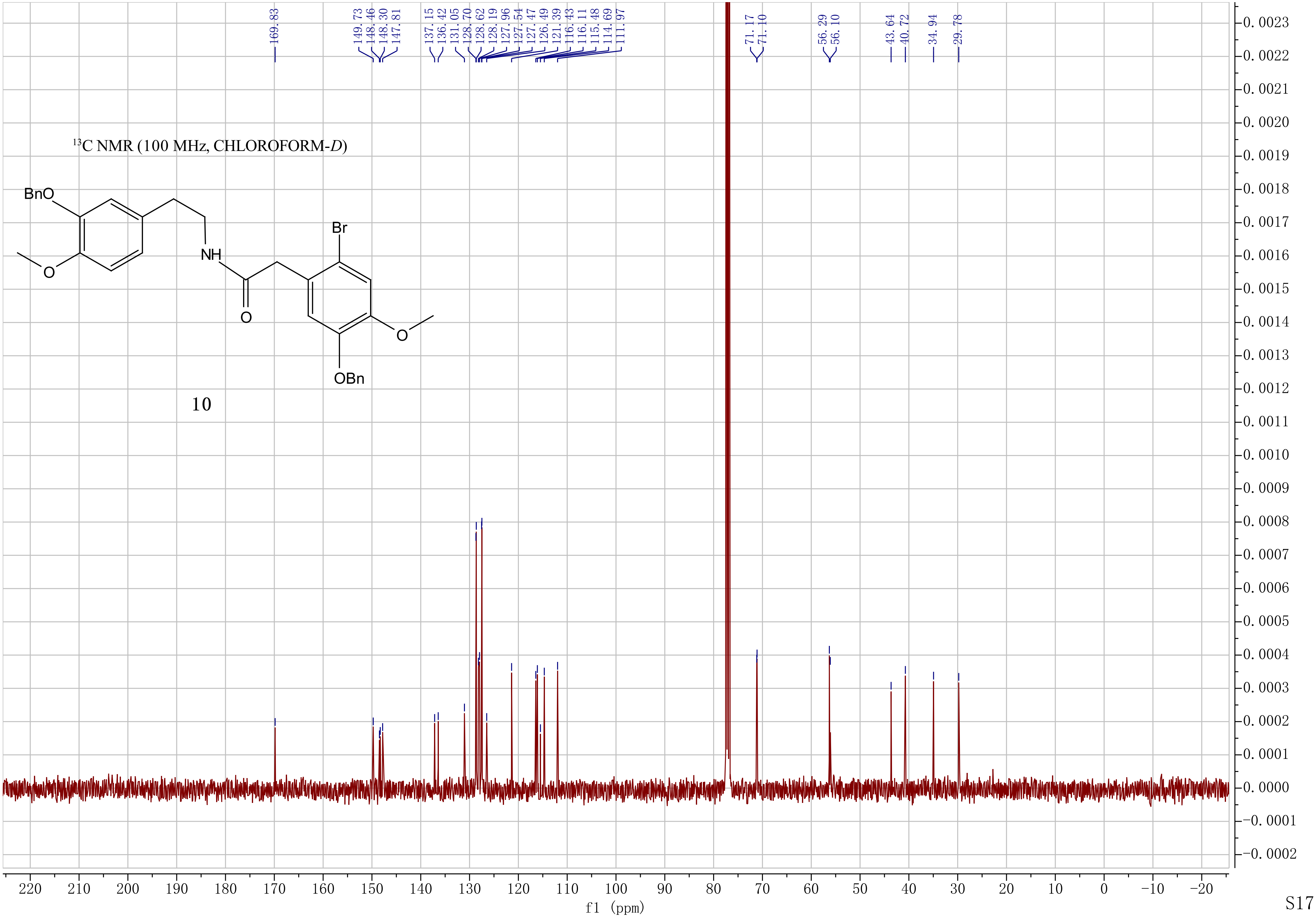

169.83
149.73
148.46
148.30
147.81
137.15
136.42
131.05
128.70
128.62
128.19
127.96
127.54
127.47
126.49
121.39
116.43
116.11
115.48
114.69
111.97
71.17
71.10
56.29
56.10
43.64
40.72
34.94
29.78
¹³C NMR (100 MHz, CHLOROFORM-D)
BnO
NH
Br
O
O
O
OBn
f1 (ppm)

10

^{1}H NMR (400 MHz, CHLOROFORM-*D*)

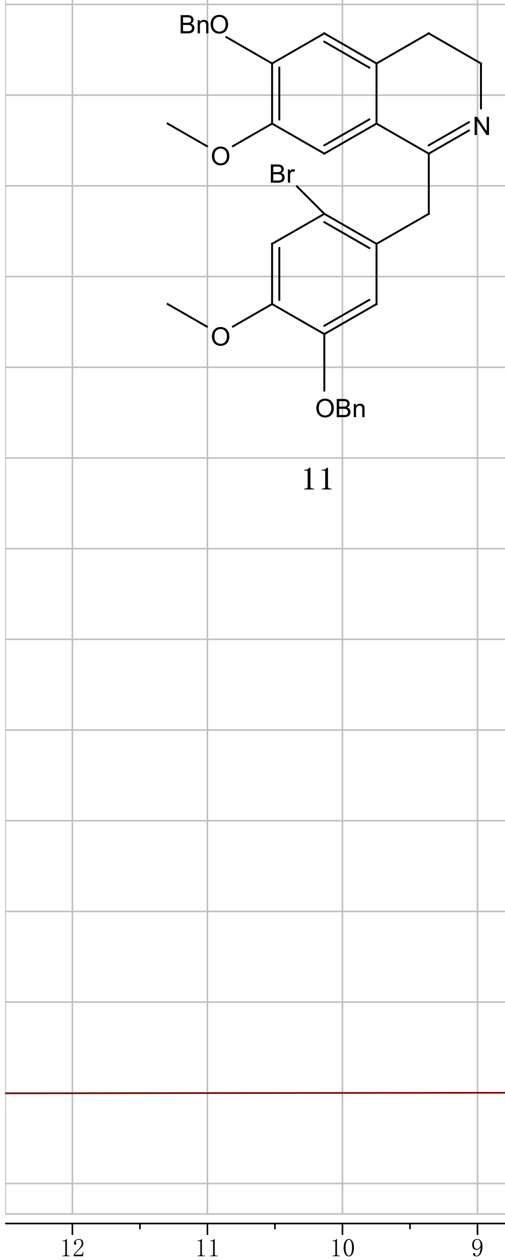

11

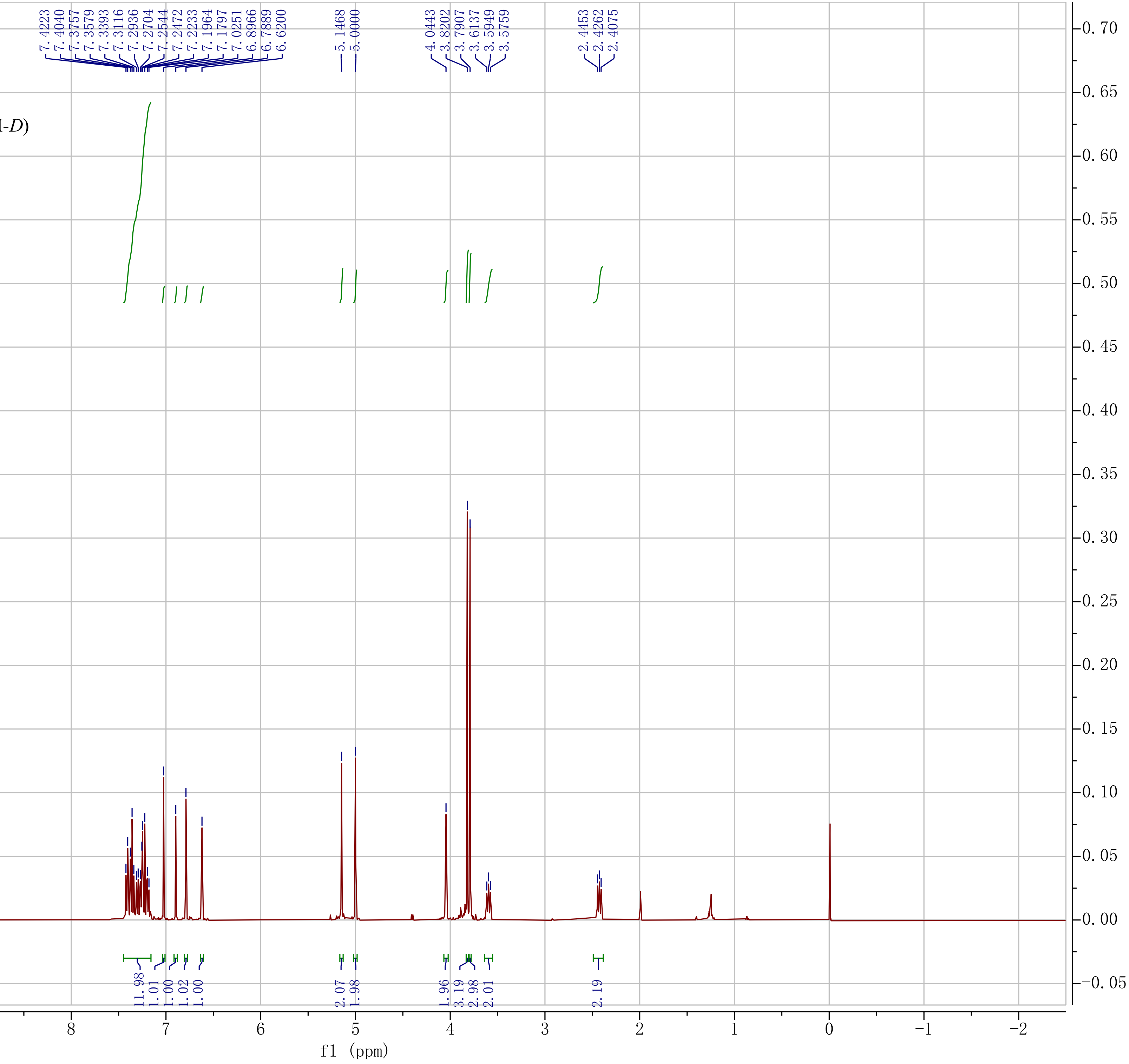

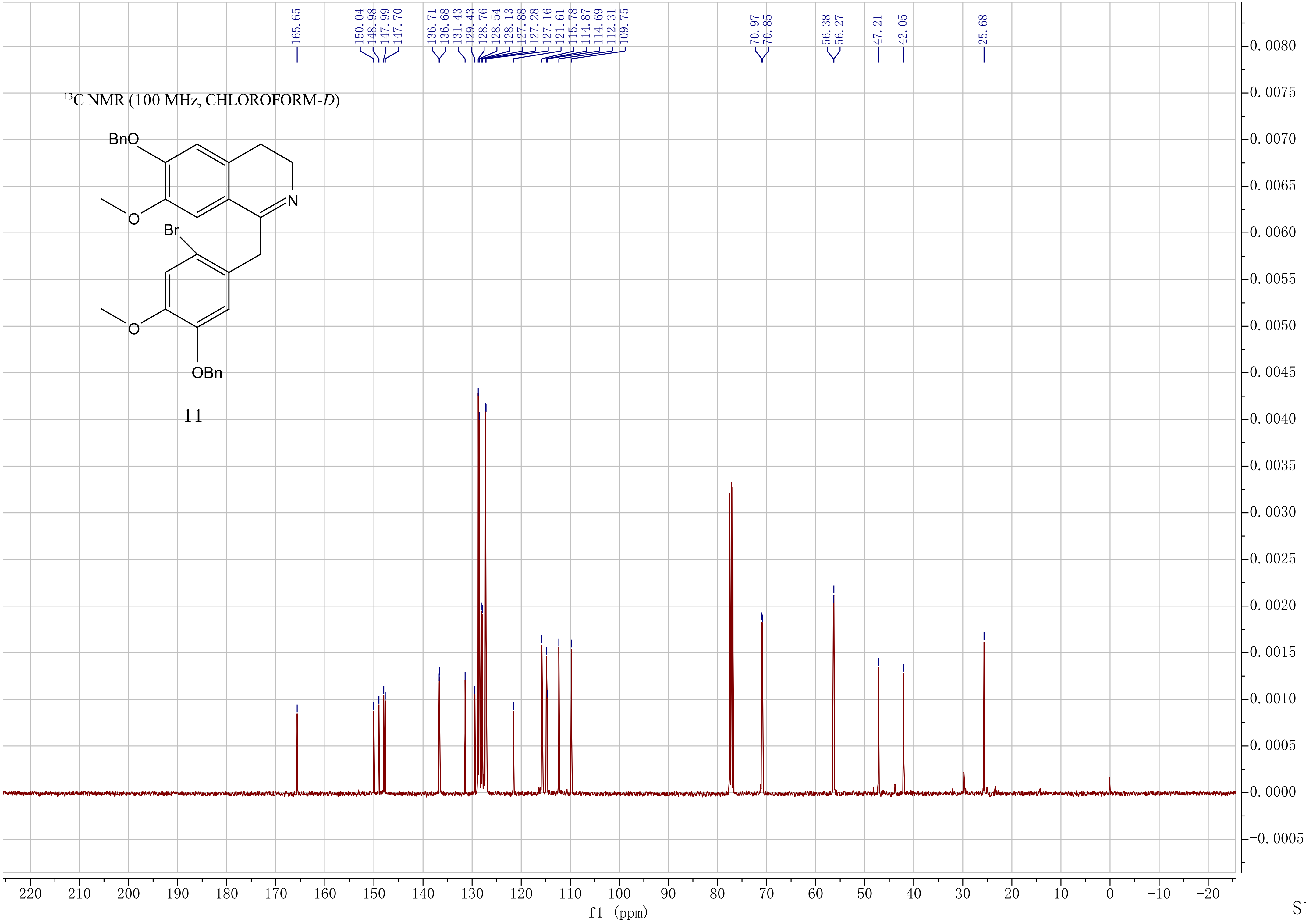

13C NMR (100 MHz, CHLOROFORM-D)
BnO
O
N
Br
O
OBn
11
165.65
150.04
148.98
147.99
147.70
136.71
136.68
131.43
129.43
128.76
128.54
128.13
127.88
127.28
127.16
121.61
115.78
114.87
114.69
112.31
109.75
70.97
70.85
56.38
56.27
47.21
42.05
25.68
f1 (ppm)

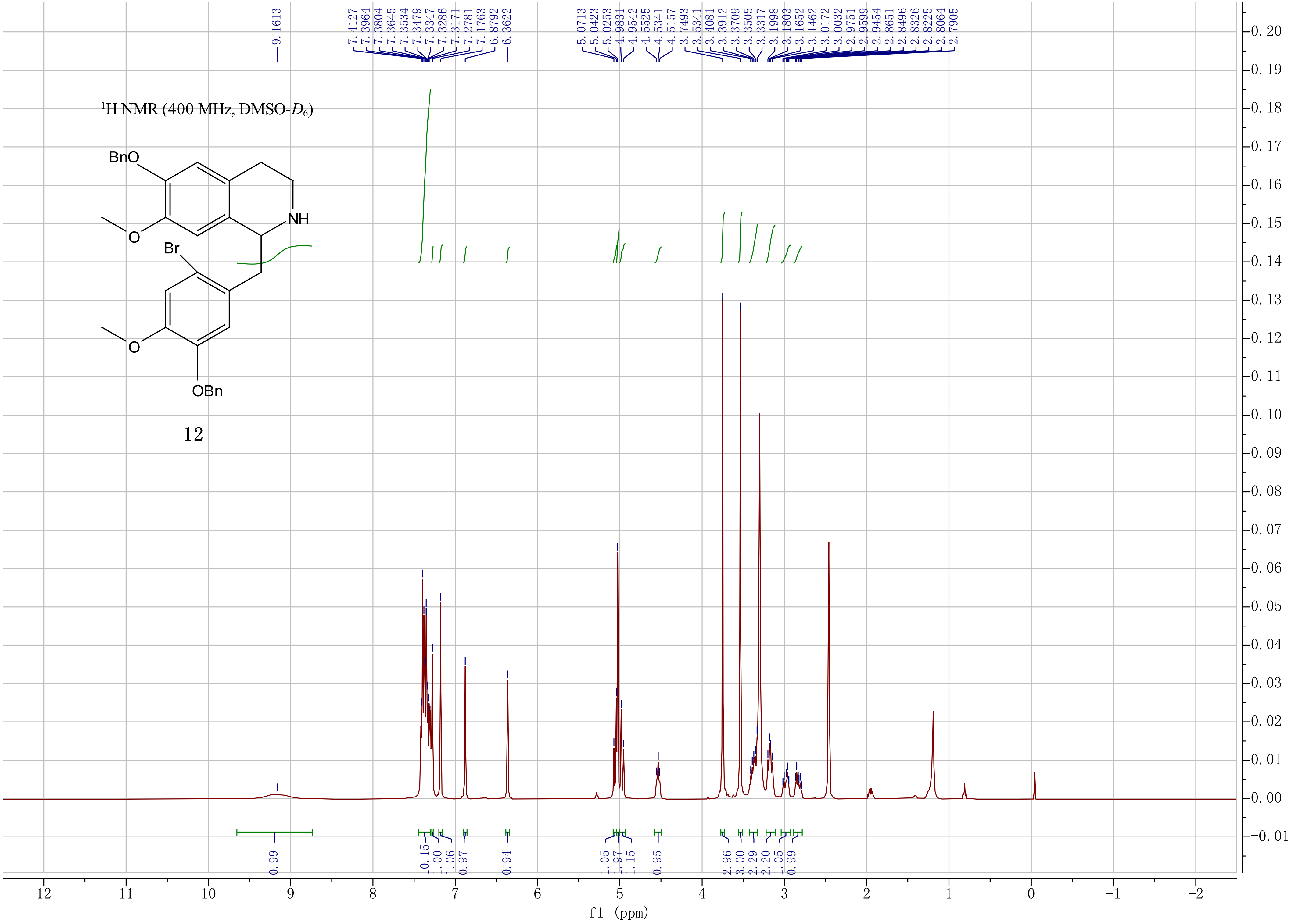

¹H NMR (400 MHz, DMSO-D6)
BnO
NH
O
Br
O
OBn
12
9.1613
7.4127
7.3964
7.3804
7.3645
7.3534
7.3479
7.3347
7.3286
7.3171
7.2781
7.1763
6.8792
6.3622
5.0713
5.0423
5.0253
4.9831
4.9542
4.5525
4.5341
4.5157
3.7493
3.5341
3.4081
3.3912
3.3709
3.3505
3.3317
3.1998
3.1803
3.1652
3.1462
3.0172
3.0032
2.9751
2.9599
2.9454
2.8651
2.8496
2.8326
2.8225
2.8064
2.7905
0.99
10.15
1.00
1.06
0.97
0.94
1.05
1.97
1.15
0.95
2.96
3.00
2.29
2.20
1.05
0.99
f1 (ppm)

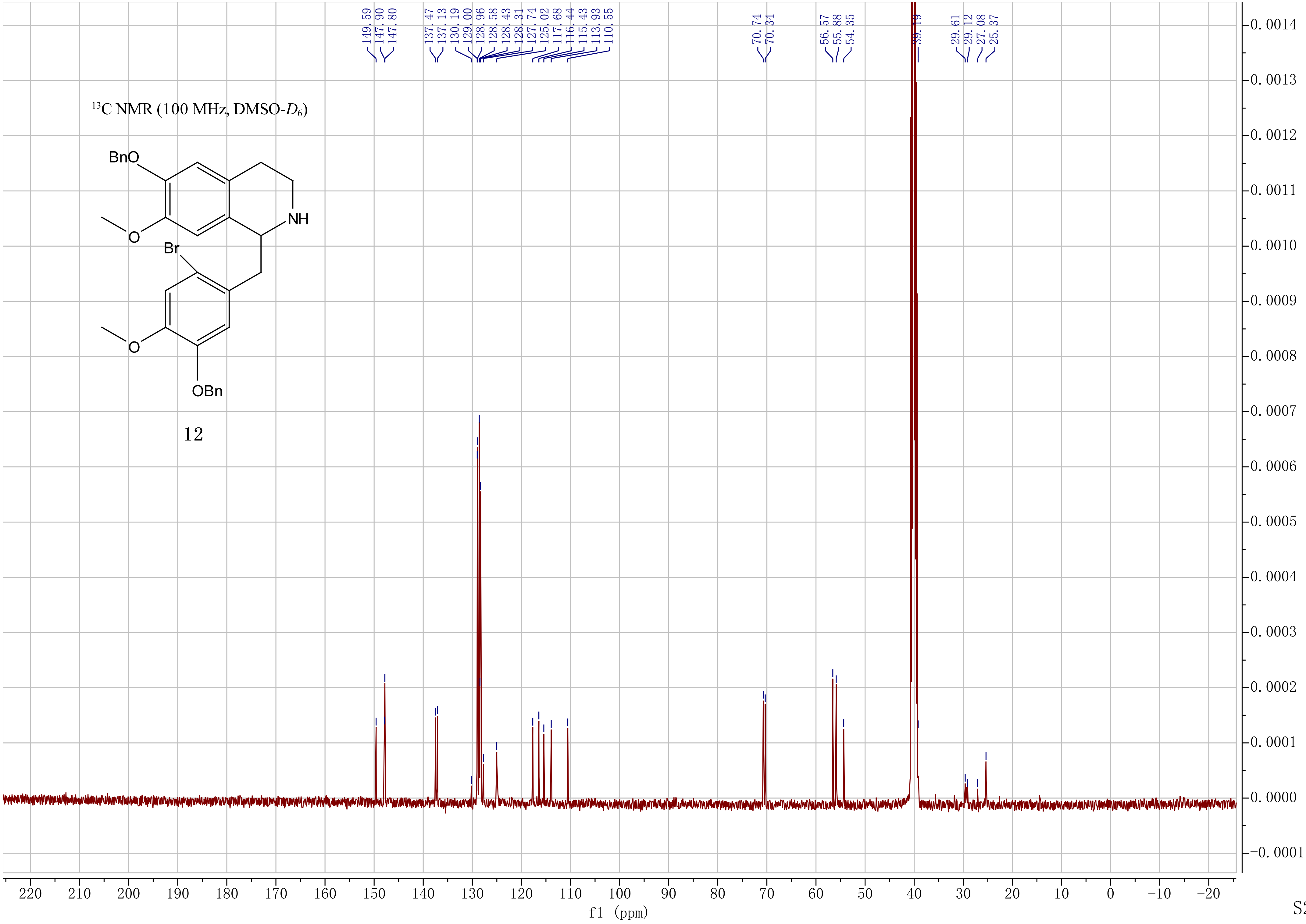

149.59
147.90
147.80
137.47
137.13
130.19
129.00
128.96
128.58
128.43
128.31
127.74
125.02
117.68
116.44
115.43
113.93
110.55
70.74
70.34
56.57
55.88
54.35
39.19
29.61
29.12
27.08
25.37
^{13}C NMR (100 MHz, DMSO-D_6)
BnO
NH
O
Br
O
OBn
12
f1 (ppm)

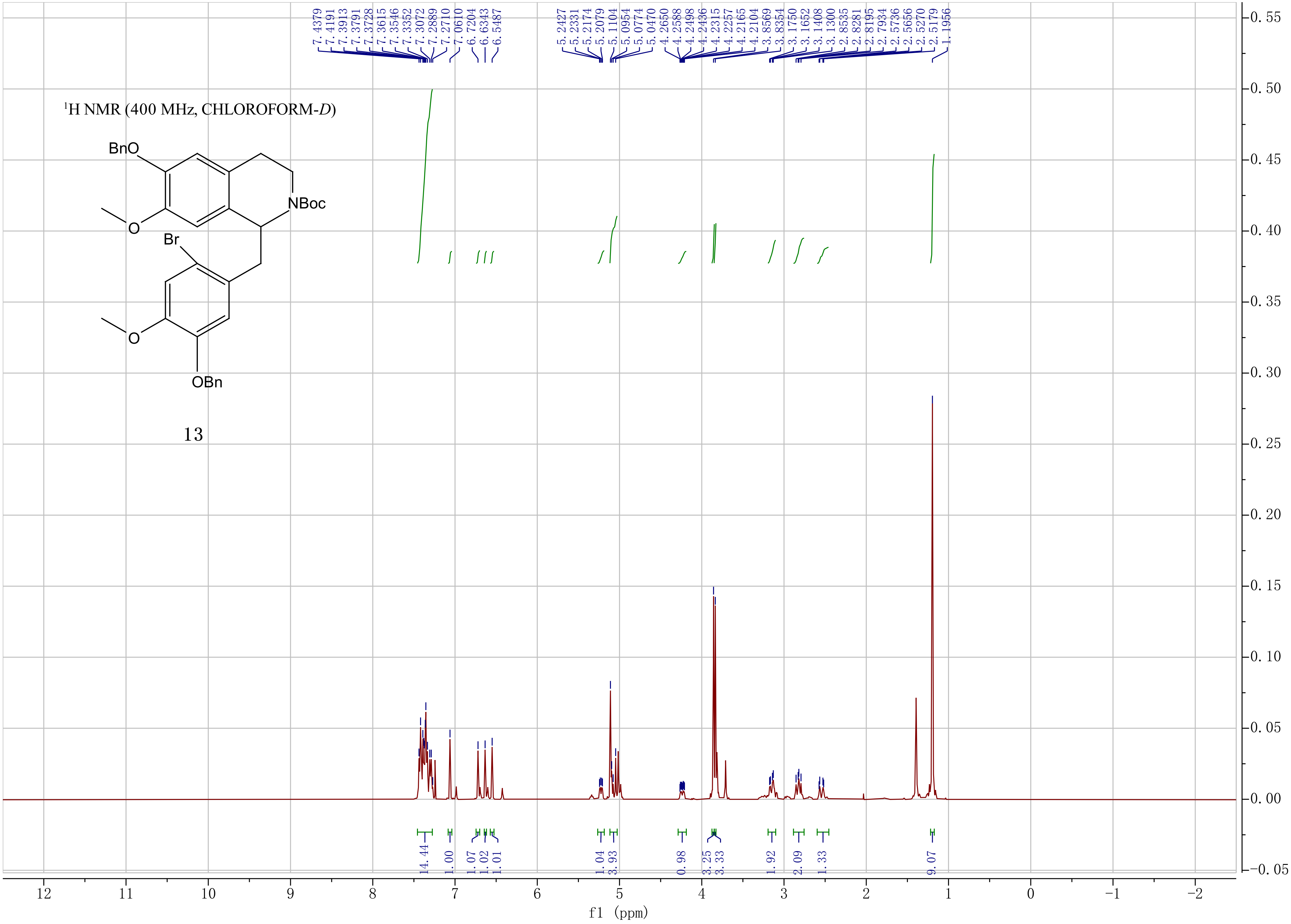

1H NMR (400 MHz, CHLOROFORM-D)
BnO
NBoc
O
Br
O
OBn
13
7.4379 7.4191 7.3913 7.3791 7.3728 7.3615 7.3546 7.3352 7.3072 7.2889 7.2710 7.0610 6.7204 6.6343 6.5487
5.2427 5.2331 5.2174 5.2079 5.1104 5.0954 5.0774 5.0470 4.2650 4.2588 4.2498 4.2436 4.2315 4.2257 4.2165 4.2104 3.8569 3.8354 3.1750 3.1652 3.1408 3.1300 2.8535 2.8281 2.8195 2.7934 2.5736 2.5656 2.5270 2.5179 1.1956
14.44 1.00 1.07 1.02 1.01 1.04 3.93 0.98 3.25 3.33 1.92 2.09 1.33 9.07
f1 (ppm)

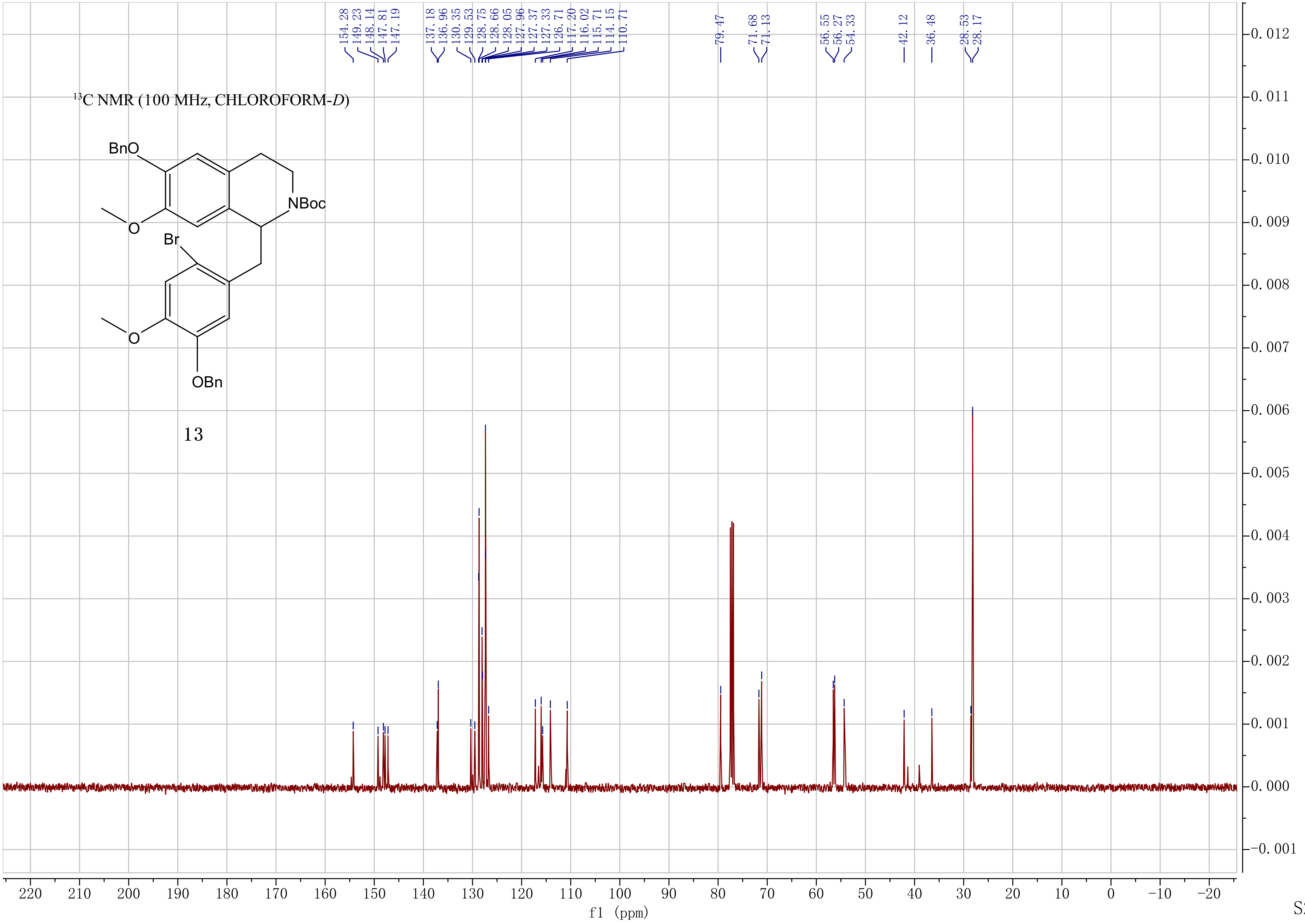
154.28
149.23
148.14
147.81
147.19
137.18
136.96
130.35
129.53
128.75
128.66
128.05
127.96
127.37
127.33
126.71
117.20
116.02
115.71
114.15
110.71
79.47
71.68
71.13
56.55
56.27
54.33
42.12
36.48
28.53
28.17
^{13}C NMR (100 MHz, CHLOROFORM-*D*)
BnO
NBoc
O
Br
O
OBn
13
f1 (ppm)

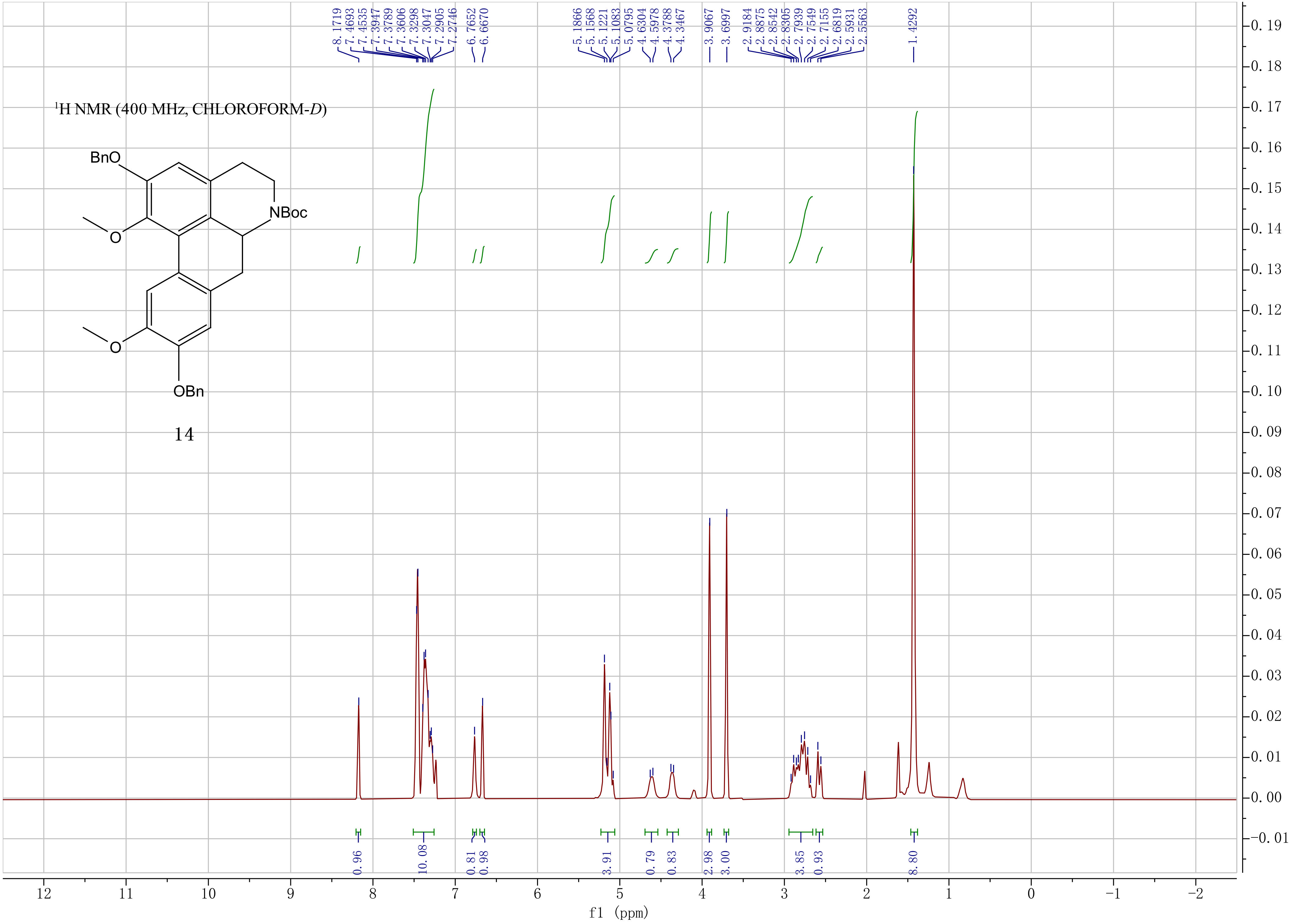

8.1719
7.4693
7.4535
7.3947
7.3789
7.3606
7.3298
7.3047
7.2905
7.2746
6.7652
6.6670
5.1866
5.1568
5.1221
5.1083
5.0795
4.6304
4.5978
4.3788
4.3467
3.9067
3.6997
2.9184
2.8875
2.8542
2.8305
2.7939
2.7549
2.7155
2.6819
2.5931
2.5563
1.4292
0.96
10.08
0.81
0.98
3.91
0.79
0.83
2.98
3.00
3.85
0.93
8.80
f1 (ppm)
^{1}H NMR (400 MHz, CHLOROFORM-*D*)
BnO
O
NBoc
O
OBn
14

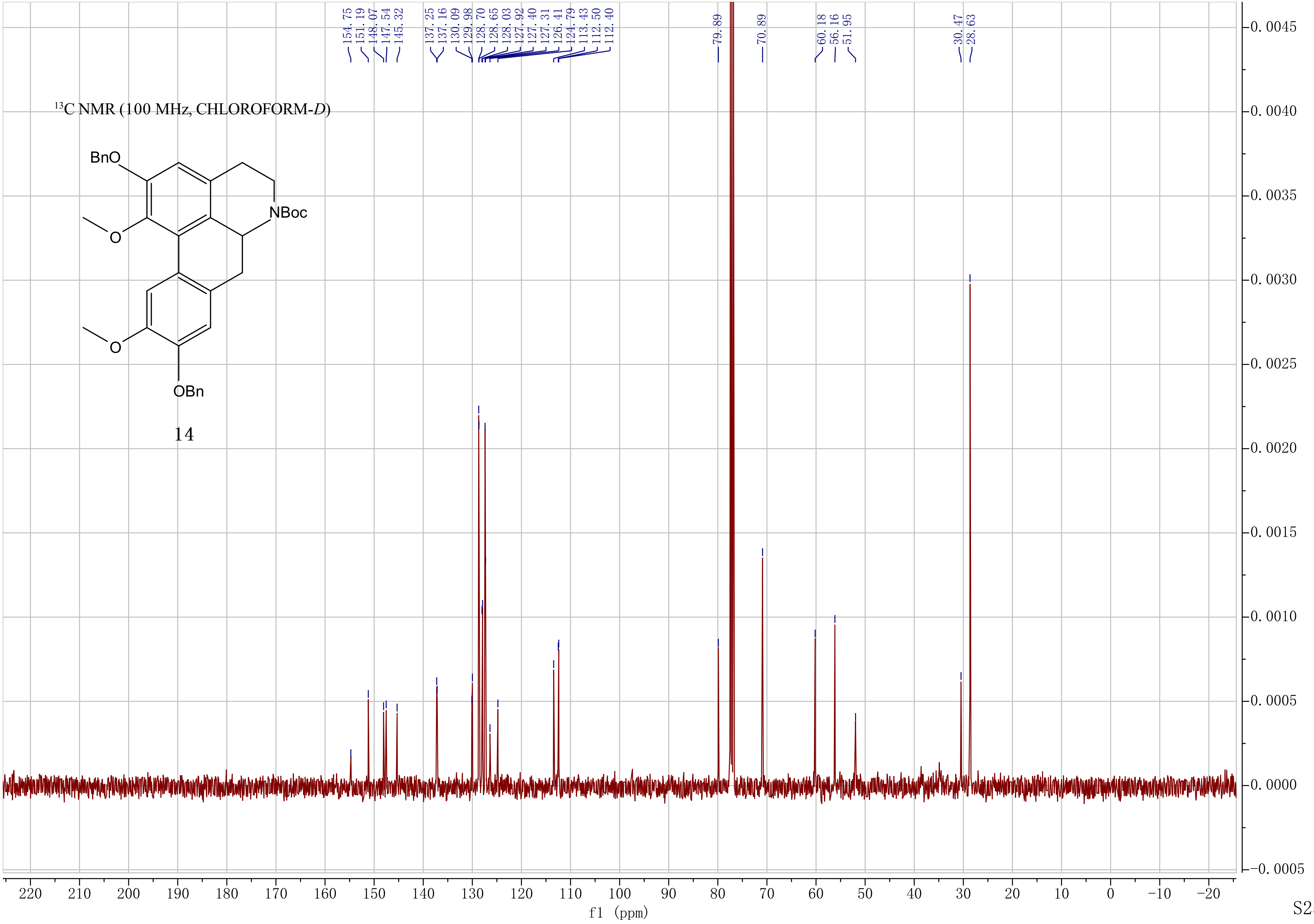

154.75
151.19
148.07
147.54
145.32
137.25
137.16
130.09
129.98
128.70
128.65
128.03
127.92
127.40
127.31
126.41
124.79
113.43
112.50
112.40
79.89
70.89
60.18
56.16
51.95
30.47
28.63
^{13}C NMR (100 MHz, CHLOROFORM-*D*)
BnO
NBoc
O
O
OBn
14
f1 (ppm)

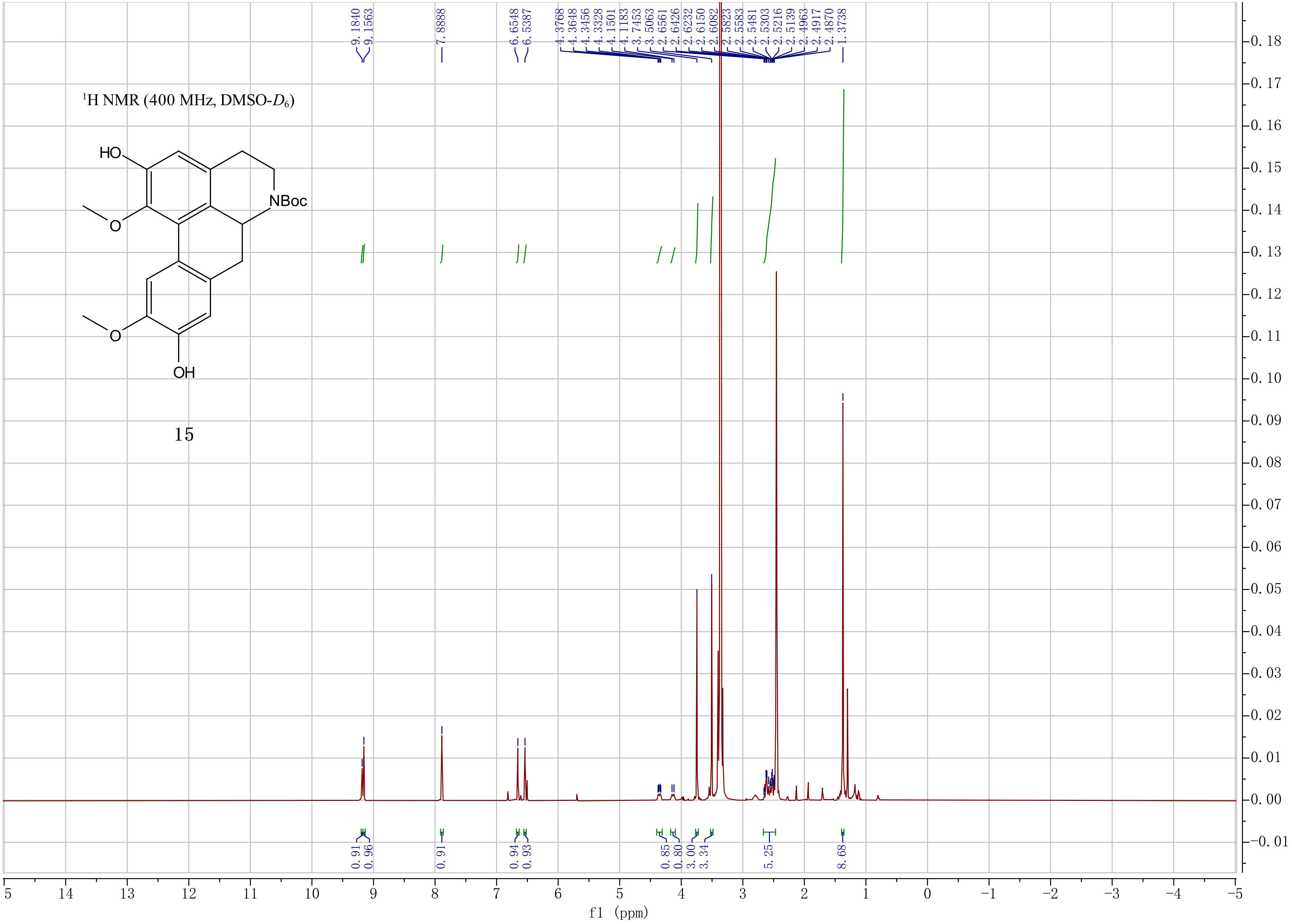

¹H NMR (400 MHz, DMSO-D_6)
HO
O
NBoc
O
OH
15
9.1840
9.1563
7.8888
6.6548
6.5387
4.3768
4.3648
4.3456
4.3328
4.1501
4.1183
3.7453
3.5063
2.6561
2.6426
2.6232
2.6150
2.6082
2.5823
2.5583
2.5481
2.5303
2.5216
2.5139
2.4963
2.4917
2.4870
1.3738
0.91
0.96
0.91
0.94
0.93
0.85
0.80
3.00
3.34
5.25
8.68
f1 (ppm)

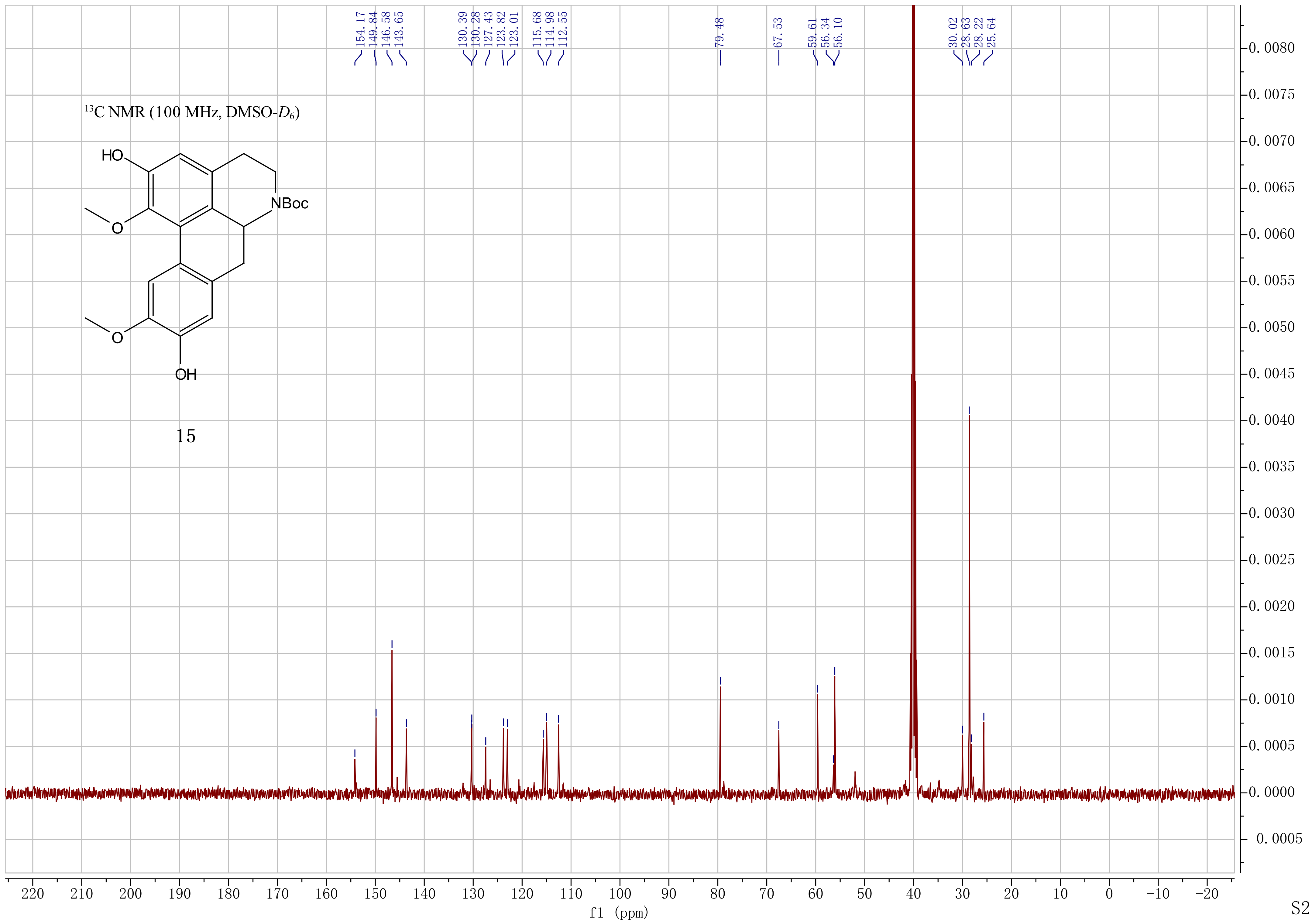

154.17
149.84
146.58
143.65
130.39
130.28
127.43
123.82
123.01
115.68
114.98
112.55
79.48
67.53
59.61
56.34
56.10
30.02
28.63
28.22
25.64
^{13}C NMR (100 MHz, DMSO-D_6)
HO
NBoc
O
O
OH
15
f1 (ppm)

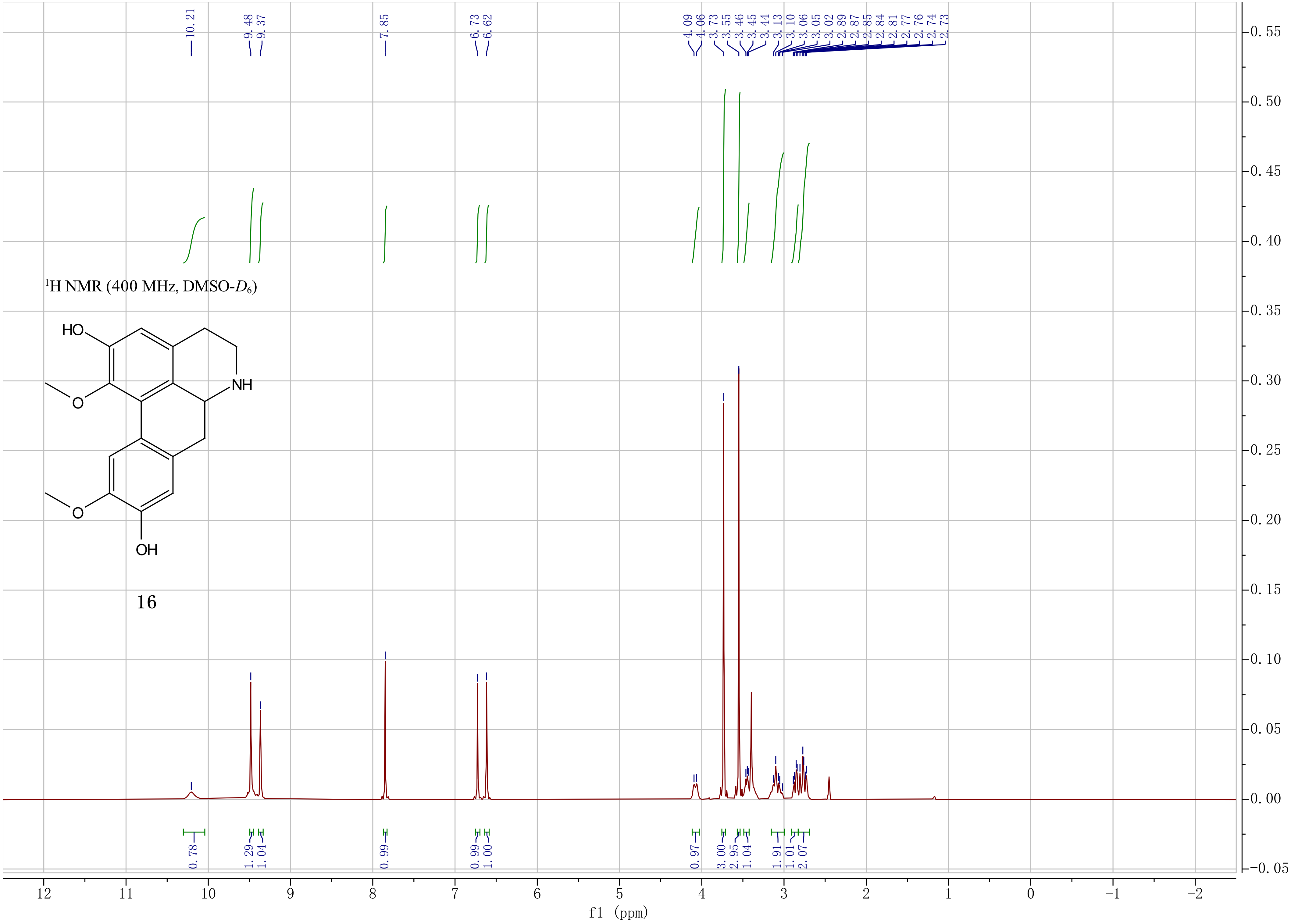

¹H NMR (400 MHz, DMSO-D_6)
HO
NH
O
O
OH
16
10.21
9.48
9.37
7.85
6.73
6.62
4.09
4.06
3.73
3.55
3.46
3.45
3.44
3.13
3.10
3.06
3.05
3.02
2.89
2.87
2.85
2.84
2.81
2.77
2.76
2.74
2.73
0.78
1.29
1.04
0.99
0.99
1.00
0.97
3.00
2.95
1.04
1.91
1.01
2.07
f1 (ppm)

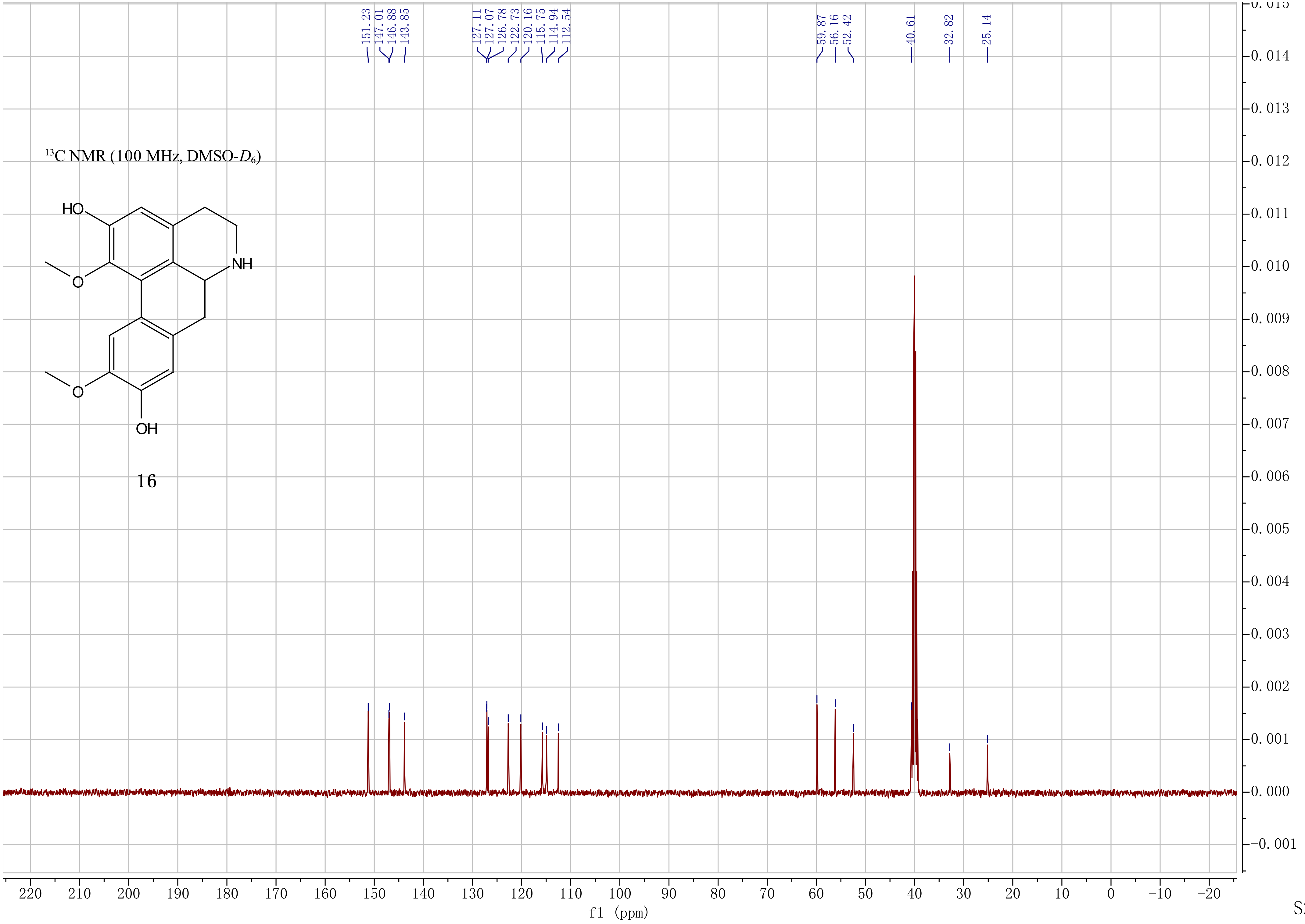
13C NMR (100 MHz, DMSO-D6)
HO
O
NH
O
OH
16
151.23
147.01
146.88
143.85
127.11
127.07
126.78
122.73
120.16
115.75
114.94
112.54
59.87
56.16
52.42
40.61
32.82
25.14
f1 (ppm)
